# TROAP regulates cell cycle and promotes tumor progression through Wnt/β‐Catenin signaling pathway in glioma cells

**DOI:** 10.1111/cns.13688

**Published:** 2021-06-02

**Authors:** Zong‐qing Zhao, Xiu‐jie Wu, Yan‐hao Cheng, Yun‐fei Zhou, Xi‐meng Ma, Jian Zhang, Xue‐yuan Heng, Fan Feng

**Affiliations:** ^1^ Department of Neurosurgery Linyi People’s Hospital Linyi China; ^2^ Institute of Brain Science and Brain‐Like Intelligence Linyi People’s Hospital Linyi China; ^3^ Institute of Clinical Medicine College Guangzhou University of Chinese Medicine Guangzhou China

**Keywords:** TROAP, glioma, cell cycle, malignant phenotype, Wnt/β‐Catenin

## Abstract

**Aims:**

Experimental evidence demonstrated a crucial role of TROAP (Trophinin‐associated protein) in regulating the cell proliferation of multiple tumors, while TROAP expression and function were largely unknown in glioma. We aimed to investigate the oncogenic role of TROAP and its potential mechanisms in gliomagenesis.

**Methods:**

Four gene expression databases (GEO, TCGA, GTEx and CCLE) were enrolled in our study and used for TROAP expression and survival analysis. TROAP expression was quantified by qRT‐PCR, western blot and immunohistochemistry assays in glioma tissues and cell lines. TROAP knockdown and overexpression vector were constructed and transfected into glioma cells. CCK‐8, colony formation, transwell, and wound healing assays were used to evaluate cell viability, migration and invasion, flow cytometry to determine cell cycle arrest. Gene set enrichment analysis (GSEA) was conducted to screen the pathway involved in TROAP‐high phenotype. The expression of cell cycle and Wnt/β‐Catenin signaling proteins were analyzed by immunofluorescence and western blot.

**Results:**

Based on the bioinformatic analysis and a series of functional assays, we found the TROAP was enriched in glioma tissues and cell lines, its overexpression was correlated with the clinicopathologic characteristics and poor prognosis. TROAP knockdown inhibited cell proliferation, migration, invasion, and G1/S cell cycle arrest compared with control group in glioma. Mechanism analysis revealed that TROAP activated Wnt/β‐Catenin pathway and upregulated its downstream targets expression, while silencing β‐Catenin or Axin2 could reverse the tumor‐promoting effects caused by TROAP, confirming that TROAP‐induced malignant phenotype and tumorigenesis via Wnt/β‐Catenin signaling pathway.

**Conclusion:**

The present study found that TROAP accelerated the progression of gliomagenesis through Wnt/β‐Catenin pathway, and TROAP might be considered as a novel target for glioma therapy.

## Graphical abstract

Trophinin‐associated protein (TROAP), a soluble cytoplasmic protein characterized by 778 amino acids, showed a cell cycle‐dependent manner, glioma cells with TROAP overexpression represented the accelerated entry into S phase and cell proliferation. TROAP, as a microtubule (MT)‐associated protein, upregulated the expression of Axin2 which disassemble the destruction complex and activated the Wnt signaling, then, β‐catenin accumulated in cytoplasm and subsequently was transported into the nucleus, which was binded with TCF to promote the transcription of downstream cell motility‐related genes, such as CyclinD1, C‐Myc, MMP7 and TCF4, inducing the process of gliomagenesis.

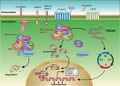

## 1 INTRODUCTION

Glioma, the most common malignant primary intracranial tumors due to carcinogenesis of brain glial cells, shows a gloomy prognosis.^1^, ^2^ Glioma, especially glioblastoma (GBM), is characterized by multifocal infiltration around the adjacent brain parenchyma. According to pathological characteristics, glioma is classified into four grade (Grade Ⅰ‐ Ⅳ), depending on which the overall survival time (OS) varies strikingly. For instance, patients with GBM (Grade Ⅳ) had a 5‐year survival rate of 5.6%, much shorter than 94.1%, the 5‐year survival rate of pilocytic astrocytoma (PA, GradeⅠ) patients.^3^ Although surgical intervention and adjuvant therapy have evolved over decades, the prognosis of patients with glioma still faces significant challenges.^4^, ^5^ Therefore, an in‐depth underlying molecular mechanisms associated with the malignant phenotype of glioma is of great value in the development of potential gene therapies.

Trophinin‐associated protein(TROAP, formally known as Tastin), a proline‐rich protein characterized by 778 amino acids, was first identified as a soluble cytoplasmic protein that participated in early embryo implantation.^6^, ^7^ A previous study has shown that TROAP was required for microtubular cytoskeleton regulation, centrosome integrity, and spindle assembly during the mitosis and cell cycle progression.^8^, ^9^, ^10^ Expect for TROAP‐induced physiological processes, several studies proved the dysregulated expression of TROAP in tumorigenesis. Results of a genomics microarray analysis indicated that TROAP presented aberrantly elevated expression in human cancer cell lines such as Jurkat and Hela cells.^11^ In recent years, aberrant expression of TROAP has been found to be responsible for the invasive behavior of various malignancies, including breast cancer, colorectal cancer, prostate cancer, gastric cancer, and hepatocellular carcinoma.^12^, ^13^, ^14^, ^15^, ^16^ However, the oncogenic functions of TROAP in astrocytoma were still unclear.

Wnt/β‐Catenin signaling pathway exerted crucial roles in many aspects of cell behavior such as proliferation, stem cell maintenance, tissue homeostasis, and cell fate decisions, imbalance in the signaling properties of Wnt/β‐Catenin could induce deregulated cell growth related to tumorigenesis.^17^, ^18^ Ye et al found that knocking down TROAP could inhibit tumor progression via activating Wnt3/survivin signaling, meanwhile induces cell cycle arrest at S phase through cyclinA2/cyclinB1 caspase pathway in prostate cancer.^14^ Several studies have reported that abnormal activation and mutation of Wnt pathway were linked to the initiation and progression of glioma.^19^, ^20^ Yet, the biological functions of TROAP and its detailed relationship with Wnt/β‐Catenin signaling were still elusive.

In our study, we aimed to ascertain TROAP expression in glioma and provide evidence for the oncogenic role of TROAP in the malignant phenotype of glioma and the underlying potential molecular mechanisms.

## 2 MATERIALS AND METHODS

### 2.1 Patients and tissue specimens

Primary glioma samples were obtained from 70 glioma patients and normal tissues were collected from 10 patients who under lobotomy after brain injure at Linyi People's Hospital (Shandong, China) between 2014 and 2019. The Department of pathology of Linyi People's Hospital elevated the tumor stage and clinical pathological classification in accordance with World Health Organization (WHO) standards. Follow‐up continued for at least 3 years or until patient death. The Ethics Committee of Linyi People's Hospital approved the present research according to the ethical guidelines of Declaration of Helsinki. Besides, informed consents were signed by each patient before experiment.

### 2.2 RNA extraction and quantitative real‐time PCR(qRT‐PCR)

RNA was harvested using the Trizol reagent (Invitrogen), according to the manufacturer's guidelines. The RNA was transcribed into cDNA by miscriptreverse transcription kit (Takara). The level of mRNA was quantified by q‐PCR with the QuantiTect SYBR Green PCR kit (Takara). The following primers were used: TROAP forward: 5‐CCTCCGGGGTGTATCTCCTAC‐3; reverse: 5‐ACGGCGCACGATGTAACAG‐3; GAPDH forward: 5‐TGACTTCAACAGCGACACCCA‐3; reverse: 5‐CACCCTGTTGCTGTAGCCAAA‐3. The expression of TROAP mRNA was normalized to GAPDH mRNA.

### 2.3 Protein preparation and western blot analysis

Tissues and cells were lysed in RIPA buffer (Beyotime) containing phosphatase inhibitors and protease inhibitors. Then, cell lysates were separated by SDS‐PAGE and the protein blots were transferred onto polyvinylidene difluoride (PVDF) membranes (Millipore). Western blot analysis was performed by the following antibodies: TROAP rabbit mAb (Proteintech, 13634‐1‐AP, 1:2000), β‐catenin rabbit mAb (No.66379‐1‐Ig, 1:4000), MMP‐7 rabbit mAb (No.1 0374‐2‐AP, 1:3000), CDK2 rabbit mAb (No.10122‐1‐AP, 1:800), CDK4 rabbit mAb (No.11026‐1‐AP, 1:3000), Axin2 rabbit mAb(No. ET1703‐96; WB 1:3000), MMP‐2 rabbit mAb (No.10373‐2‐AP, 1:3000), RHOA rabbit mAb (No.10749–1‐AP, 1:2000), ROCK1 rabbit mAb (No.21850‐1‐AP, 1:2000), GAPDH mouse mAb(No.60004‐1‐Ig, 1:3000).

### 2.4 Immunohistochemical staining

Paraffin‐embedded sections (thickness, 3 μm) were dehydrated by a graded series of ethanol and subjected to antigen retrieval, an UltraSensitive SP IHC kit (Maxin) was used for immunohistochemical staining. The sections were incubated with anti‐TROAP antibody at dilution of 1:200 (Proteintech) at 4℃ overnight. Then, the sections were developed with DAB and counterstained with hematoxylin. The scoring standards for staining intensity for TROAP were as follows: the percentage of positive cell scores: [score 0, no staining; score 1, <10%; score 2, 10%–30%; score 3, >30%]. The staining intensity score: [score 0, negative; score 1, weak intensity; score 2, moderate intensity; score 3 strong intensity]. Sum score <4 means negative and sum scores >=4 was considered as positive.

### 2.5 Cell culture and transfection

Cell lines, including glioma cell lines (A172, SF295, TJ905, PT2, U251) and normal human astrocyte cell line (HA1800), were purchased from Shanghai Cell Bank of Chinese Academy of Science (Shanghai, China). Cells were cultured in Dulbecco's Modified Eagle Medium (DMEM) with 10% fetal bovine serum (FBS), streptomycin (10 μg/ml) and penicillin (100 U/ml) in a 5% CO2 incubator at 37℃. The sequence of TROAP siRNA‐1, siRNA‐2, siRNA‐3 and si‐NC were as follows: siRNA‐1 forward: 5‐GCAGAAACCACCGCUCAAUTT‐3, reverse: 5′‐AUUGAGCGGUGGUUUCUGCTT‐3′; siRNA‐2 forward: 5′‐CCAACCCUGUGGCUACAUUTT‐3′, reverse: 5′‐AAUGUAGCCACAGGGUUGGTT‐3′; siRNA‐3 forward: 5′‐CCUCUUAAUGGAGGCUCUUTT‐3′, reverse: 5′‐AAGAGCCUCCAUUAAGAGGTT‐3′; si‐NC forward: 5′‐UUCUCCCGAACGUGUCACGUTT‐3′, reverse: 5′‐ACGUGACACGUUCGGAGAATT‐3′. To overexpress TROAP, the coding sequence was obtained from NCBI and synthesized to facilitate colony. Lentivirial particles were purchased at Genechem. Co. Ltd. Transfected cells were screened by polybrene and used for subsequent experiments.

### 2.6 CCK‐8 and colony formation assay

For CCK‐8 assay, cells were cultured in 96‐well plates (3 × 10^3^/well) with 100 μl medium per well. After culture for 24, 48, 72, and 96 h, cell counting kit 8 (CCK‐8) was added into each well and then the absorbance was measured at a wavelength of 450 nm.

For colony formation assay, cells were seeded into 6‐well plates (1 × 10^3^/well) and incubated for 14 days until visible clones appeared, Then, cells were fixed under 4% methyl alcohol for 20 min and stained using 5% crystal violet for 15 min. Finally, clones were counted under a microscope.

### 2.7 Wound healing and transwell assays

For wound healing assay, cells were seeded in 6‐well plates and wounds were made with yellow pipette tips, then the cell monolayers were washed twice with PBS. Images of the width of wound were photographed at 0, 12, 24, 36, and 48 h later.

For transwell assay, the experiment was performed with the 8μm‐pore chamber inserted into 24‐well plates, 5 × 10^5^ transfected cells were placed into upper chamber coated with Matrigel (Dilution 1:7). DMEM containing 10% FBS was added to the lower chamber. After 24 h incubation, cells that had invaded through the membrane were fixed with 4% paraformaldehyde and stained with 0.2% crystal violet for 15 min, counted and photographed with an inverted microscope.

### 2.8 Cell cycle analysis

Cells were seeded in 6‐well plates at a destiny of 5 × 10^5^ cells /well and cultured to 70%–80% confluence. Cells were harvested and washed twice with ice‐cold PBS, then fixed with 70% cold ethanol at 4% overnight and incubated with RNase and propidium iodide (PI). Cell cycle was detected by EPICS XL Flow Cytometer (Beckman, Coulter) and Modfit software was used for data analysis.

### 2.9 Immunofluorescence Analysis

Cells grown on coverslips were fixed in 4% paraformaldehyde and then permeabilized by incubation with TritonX‐100 (Sigma) for 15 min. After washing twice with PBS, cells were blocked with 2% BSA and incubated with anti‐Axin2 (1:200, Huabio) and anti‐β‐Catenin (1:200 Proteintech) at 4℃ overnight. Then, coverslips were incubated with TRITC‐conjugated secondary antibody (1:200, Affinity) at room temperature for 30 min, washed with PBS, and incubated with DAPI for 30 min at 37℃. Fluorescence was monitored by Olympus fluorescence microscope.

### 2.10 Microarray data preparation

The raw data of six glioma microarray datasets, including GSE4412, GSE16011, GSE52009, GSE501161, GSE4290 and GSE67809, were downloaded from the GEO database (https://www.ncbi.nlm.nih.gov/geo/). The TCGA, CCLE, GTEx RNA‐seq data of glioma were obtained from UCSC Cancer Browser (https://xenabrowser.net/).

### 2.11 Bioinformatic analysis

Gene set enrichment analysis was used to generate an ordered lists of all genes related to the level of TROAP. Then, GSEA was performed to identify differentially enriched biological pathways between the high and low TROAP groups. The expression of TROAP was used as a phenotype label. The normalized enrichment score (NES) and false discovery rate (FDR) were performed to sort enriched biological pathways in each phenotype. Moreover, other R packages including “ggpubr”, “ggplot2”, “limma” etc, were applied for visualizing the results of bioinformatic analysis.

### 2.12 Statistical analysis

Statistical analysis was performed using Origin 2018 and Sigmaplot version 14.0 for Windows. Data were expressed as the Means ± SD. Student's *t*‐test was used to analyze differences between two groups. Comparisons between more than three groups were determined using one‐way ANOVA analysis of variance followed by the Turkey post hoc test. The χ^2^ test examines the relationship between TROAP and clinicopathological characteristics. Kaplan‐Meier survival analysis was used to analyze overall survival (OS) time. A value of *p *< 0.05 was considered statistically significant.

## 3 RESULTS

### 3.1 Expression patterns of TROAP in glioma tissue and cell lines

**FIGURE 1:**
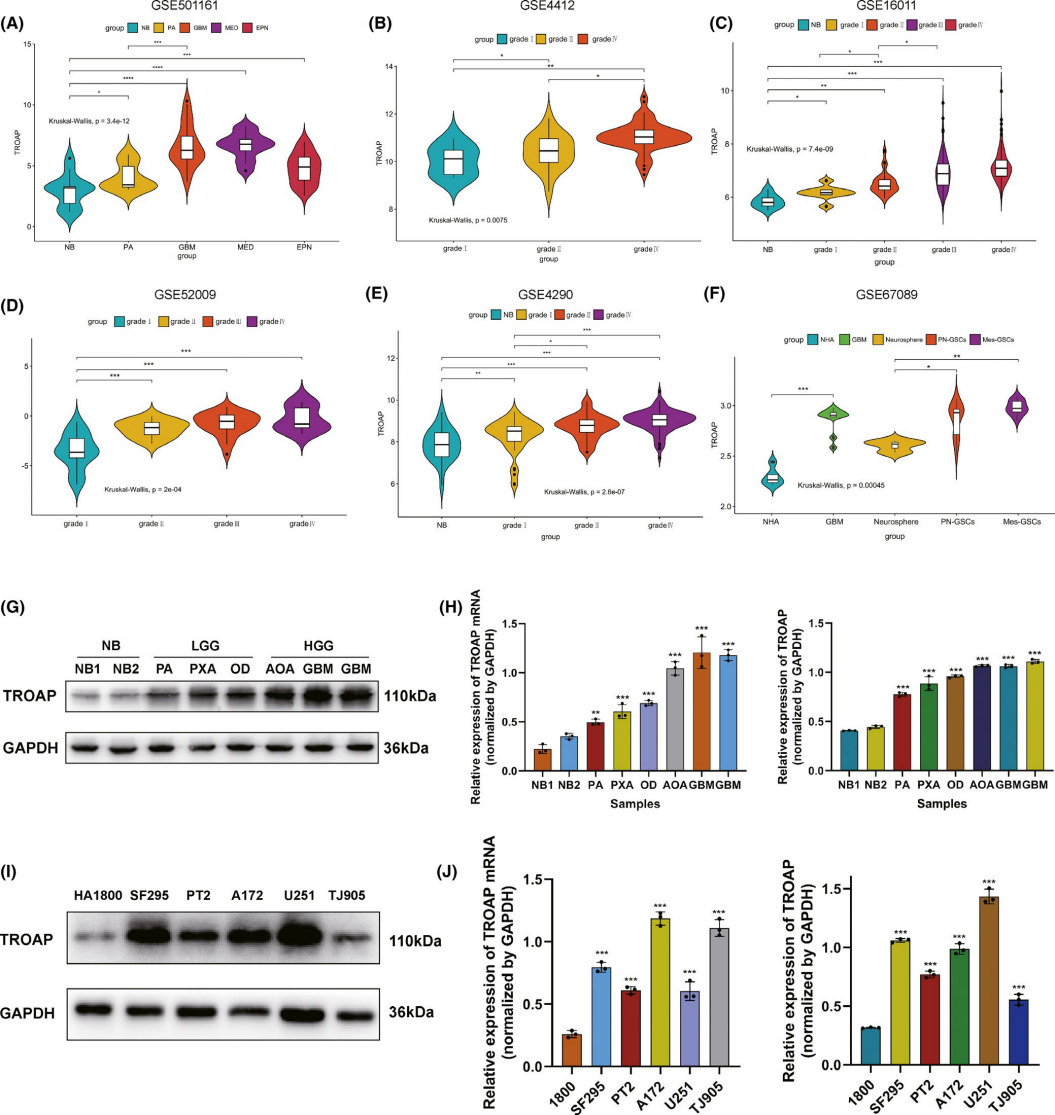
High level of TROAP expression correlated with the progressive malignancy in glioma. (A) Expression patterns of TROAP in several types of CNS tumors from GEO database. (B–E) In GEO database, TROAP was markedly increased in high‐grade glioma. (F) Compared with the normal astrocyte and neuro stem cells (NSCs), TROAP expression was upregulated in glioblastoma cells and GSCs (especially in Mes‐GSCs). (G–H) Western blot and qRT‐PCR analysis of TROAP in non‐tumor brain and glioma tissues collected from Linyi People's Hospital. (I–J) mRNA and protein level of TROAP in human astrocyte cell lines and five glioma cell lines measured by qRT‐PCR and western blot. (LGG, low‐grade glioma; HGG, high‐grade glioma; PA, Pilocytic astrocytoma; PXA, Pleomorphic xanthoastrocytoma; OD, oligodendroglioma; AOA, Anaplastic Oligodendroglioma; GBM, Glioblastoma Multiforme; *p < 0.05, **p < 0.01, ***p < 0.001, respectively)

To elucidate the alteration of TROAP expression among multiple types of tumors, we initially analyzed the different mRNA levels of TROAP in several types of central nervous system (CNS) tumors and found that the expression of TROAP mRNA was higher in glioma (GSE501161, Figure 1A). Meanwhile, analysis using the GEO datasets (GSE4412, GSE16011, GSE52009, GSE4290, Figure 1B–E) indicated that the expression of TROAP mRNA was significantly increased in glioma tissues, especially in GBM, compared to normal brain tissue. Besides, TROAP indicated significant hyper‐expression in glioblastoma stem cells (GSCs, especially in mesenchymal GSCs) and GBM cells compared with neuro stem cells (NSCs) and normal human astrocyte (NHA), suggesting that TROAP had the potential to promote the malignant phenotype of glioma (GSE67089, Figure 1F). Then, the results of qRT‐PCR and western blot assays revealed that in comparison with control group, TROAP expression was markedly upregulated with increasing WHO grade in glioma samples (*p *< 0.05, Figure 1G,H). In addition, we also measured TROAP mRNA and protein level in a normal astrocyte cell line (HA1800) and 5 glioma cell lines (U251, SF295, TJ905, A172, PT2). Findings showed that TROAP expression was higher in glioma cell lines (especially U251 and SF295) compared to normal astrocyte cells (*p *< 0.05, Figure 1I,J).

### 3.2 TROAP overexpression was associated with clinical features and poor prognosis

**FIGURE 2:**
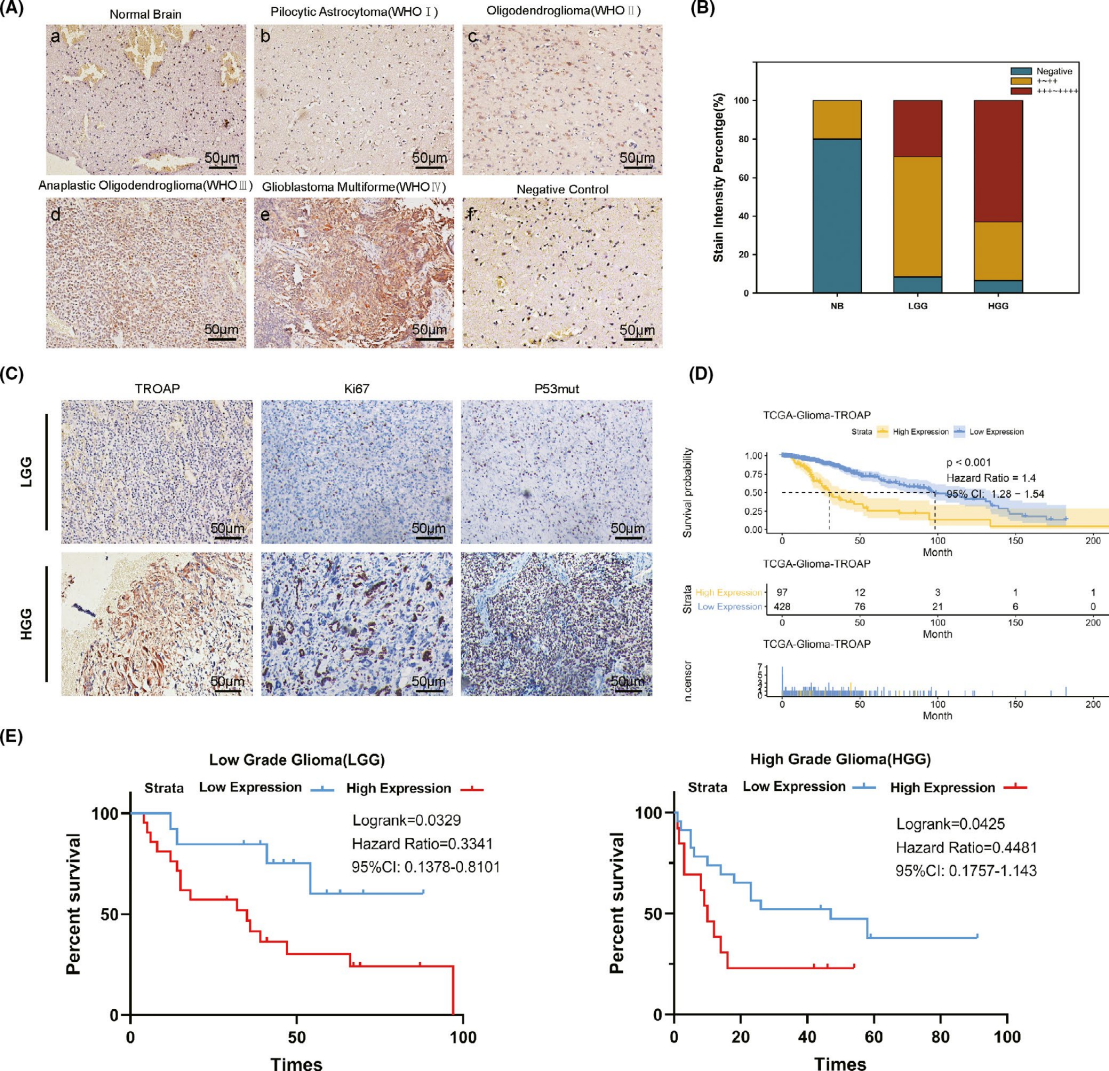
TROAP was overexpressed in glioma tissues and correlated with overall survival. (A, B) Immunohistochemical staining of TROAP in glioma (WHO grade Ⅰ‐ Ⅳ) and non‐tumor tissues (a, non‐tumor, *n* = 10; b, gradeⅠ, *n* = 4; c, gradeⅡ, *n* = 20; d, gradeⅢ, *n* = 32; e, gradeⅣ, *n* = 14; f, negative control) scale bar, 50 μm. (C) IHC assay of TROAP and malignant tumor biomarkers (Ki‐67, P53mut) in patients with different grade of glioma. (D, E) Kaplan‐Meier analysis for overall survival (TCGA, *n* = 525, Linyi People's Hospital, LGG, *n* = 24; HGG, *n* = 46) in glioma patients according to TROAP expression level (Log‐rank test, *p < 0.05, **p < 0.01, ***p < 0.001, respectively)

We reviewed the clinical characteristics of 70 patients with glioma and explore the association between TROAP expression and the clinicopathological features via immunohistochemistry assay (Table 1). Findings represented weak/negative staining for TROAP in normal brain sample, moderate to strong cytoplasmic staining for TROAP in low‐grade glioma (such as Pilocytic astrocytoma, Pleomorphic xanthoastrocytoma and Oligodendroglioma) and high‐grade glioma (such as Anaplastic Oligodendroglioma and Glioblastoma Multiforme) tissues (Figure 2A,B). Overexpression of TROAP protein was significantly correlated with WHO grade, Ki67 and P53mut (Figure 2C, Table 2). To further investigate the potential role of TROAP in glioma, we performed Kaplan‐Meier analysis of overall survival time (OS) in the TCGA glioma database and found that patients stratified by a media cutoff of TROAP level with lower TROAP expression had longer OS than those with higher levels of TROAP (Figure 2D). Similarly, we also analyzed the TROAP expression profiles of patients (LGG and HGG) involved in our study for whom OS data were available, and the results were consistent with the conclusion from TCGA database, suggesting the high level of TROAP indicated poor prognosis of patients with glioma (Figure 2E).

**TABLE 1:** Immunohistochemical analysis of TROAP in glioma tissue

Group	Case	Score of TROAP expression		
		Negative	Low	High
NB	10	8 (80.0)	2 (20.0)	0 (0)
Grade Ⅰ‐Ⅱ	24	2 (8.3)	15 (62.5)	7 (29.2)
Grade Ⅲ‐Ⅳ	46	3 (6.5)	14 (30.4)	29 (63.1)

**TABLE 2:** Expression of CUX1 in relation to clinicopathological features

Clinicopathological Features value	TROAP expression			
	*N*	Low expression	High expression	*p* value
Gender				
Male	32 (45.7)	18 (56.3)	14 (43.7)	
Female	38 (54.3)	14 (36.8)	24 (63.2)	0.104
Age				
<44	28 (40.0)	10 (35.7)	18 (64.3)	
≥44	42 (60.0)	22 (52.4)	20 (47.6)	0.170
Tumor diameter				
<4 cm	16 (22.9)	6 (37.5)	10 (63.5)	
≥4 cm	54 (78.1)	26 (48.1)	28 (51.9)	0.453
Tumor location				
Frontal	36 (51.4)	15 (41.7)	21 (58.3)	
Temporal	20 (28.6)	10 (50.0)	10 (50.0)	
Other	14 (20.0)	7 (50.0)	7 (50.0)	0.783
KPS				
<80	35 (50.0)	12 (34.3)	23 (65.7)	
≥80	35 (50.0)	20 (57.1)	15 (42.9)	0.055
WHO grade				
Ⅰ‐Ⅱ	24 (34.3)	17 (70.8)	7 (29.2)	0.002**
Ⅲ‐Ⅳ	46 (65.7)	15 (32.6)	31 (67.4)	
P53mut expression				
Low	44 (62.9)	28 (63.6)	16 (36.4)	0.008**
High	26 (39.1)	8 (30.8)	18 (69.2)	
Ki−67 expression				
Low	30 (42.9)	21 (70.0)	9 (30.0)	0.0004***
High	40 (57.1)	11 (27.5)	29 (72.5)	

### 3.3 TROAP knockdown inhibited glioma cell proliferation by inducing G1/S cell cycle arrest

**FIGURE 3:**
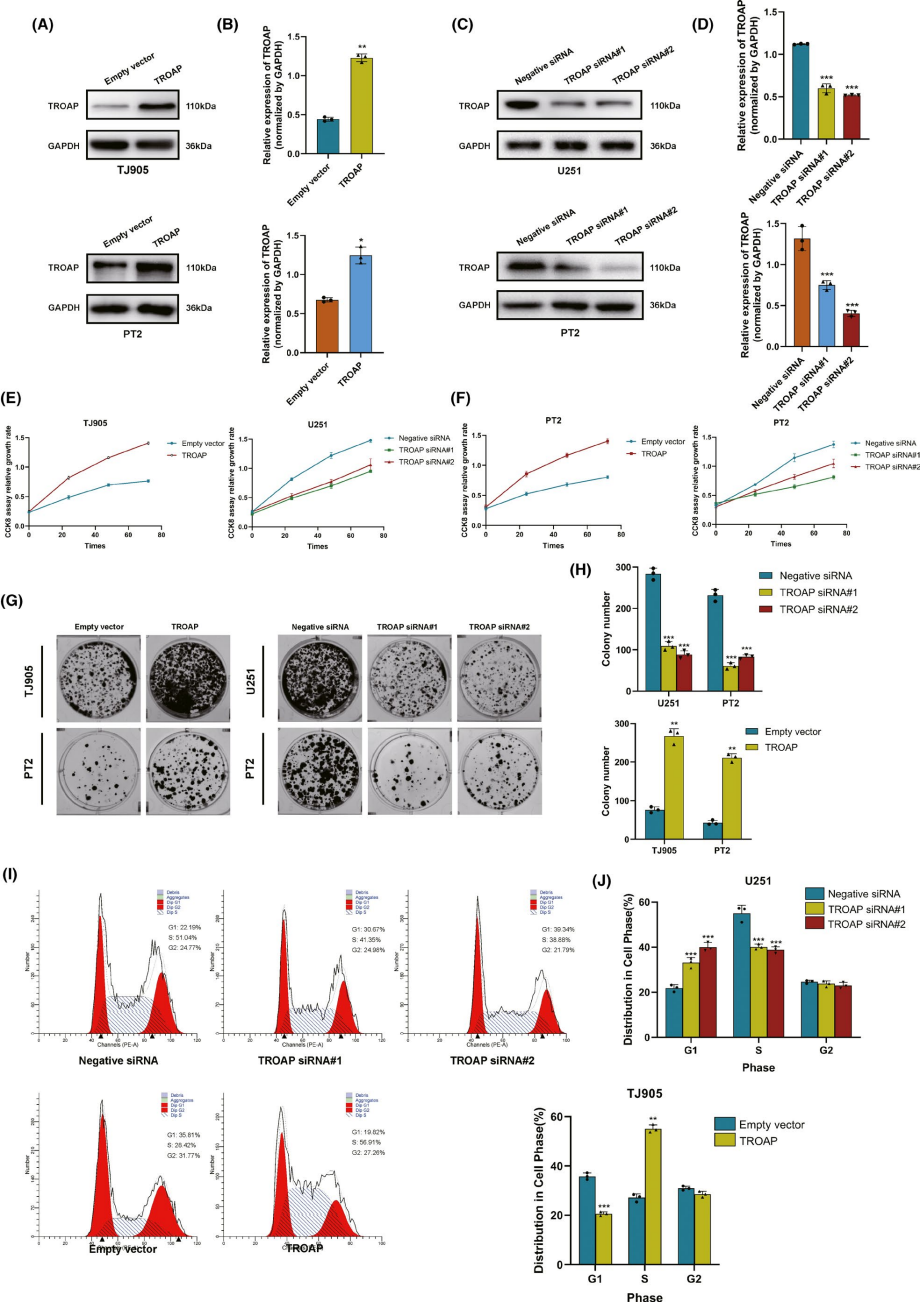
TROAP knockdown inhibited glioma cell proliferation by inducing G1/S cell cycle arrest. (A‐D) The interference effects of TROAP siRNA and overexpression plasmid in U251, TJ905 and PT2 cells. (E‐F) The proliferation rate of U251, TJ905 and PT2 cells transfected with TROAP siRNA and plasmid determined by CCK8 assay. (G, H) Colony formation assay of U251, TJ905 and PT2 cells treated with TROAP siRNA and overexpression plasmid after 21 days. The statistics were shown in the histogram. (I, J) Flow cytometry data represented more cells were accumulated in S phase of cell cycle in the TROAP overexpression group compared with empty vector group, while TROAP knockdown induced G1 phase arrest of glioma cells. Data were presented as mean ± SD of three of separate experiments (*p < 0.05, **p < 0.01, ***p < 0.001, respectively)

To investigate the effect of TROAP on biological behavior of glioma, pSIN‐TROAP‐derived lentivirus was used to transfect TJ905 glioma cell lines to establish TROAP‐over‐expression stable cell lines, besides, U251 cell lines were transfected with TROAP‐siRNA vector. The transfection efficiency assays were performed to detect the function of TROAP in glioma cells (Figure 3A,B). CCK‐8 and colony formation assays showed that the viability of U251 and TJ905 cells was significantly lower and higher after transfection with TROAP‐siRNA and TROAP overexpression vector, respectively (Figure 3E,G–H). Different glioma cell lines exhibit different phenotypes and properties. To eliminate potential interference by different cell line phenotypes, we also selected the PT2 cell line for knockdown and overexpression transfection assays, followed by a series of cellular function assays (*p *< 0.05, Figure 3C,D). These observations were also consistent with the results of parallel CCK8 and colony formation assays in the PT2 cell line (*p *< 0.05, Figure 3F–H). These data suggested that TROAP facilities proliferation of glioma cells. Then, the role of TROAP in cell cycle progression was explored, flow cytometry studies showed that TROAP overexpression promoted the accumulation of TJ905 cells in S phase compared to control, while TROAP knockdown significantly increased the percentage of U251 cells in G1 phase compared to control (*p *< 0.05, Figure 3I,J). Hence, we speculated that TROAP knockdown inhibited glioma cell proliferation by inducing G1/S cell cycle arrest.

### 3.4 Upregulated TROAP promoted glioma cells invasion and migration in vitro

**FIGURE 4:**
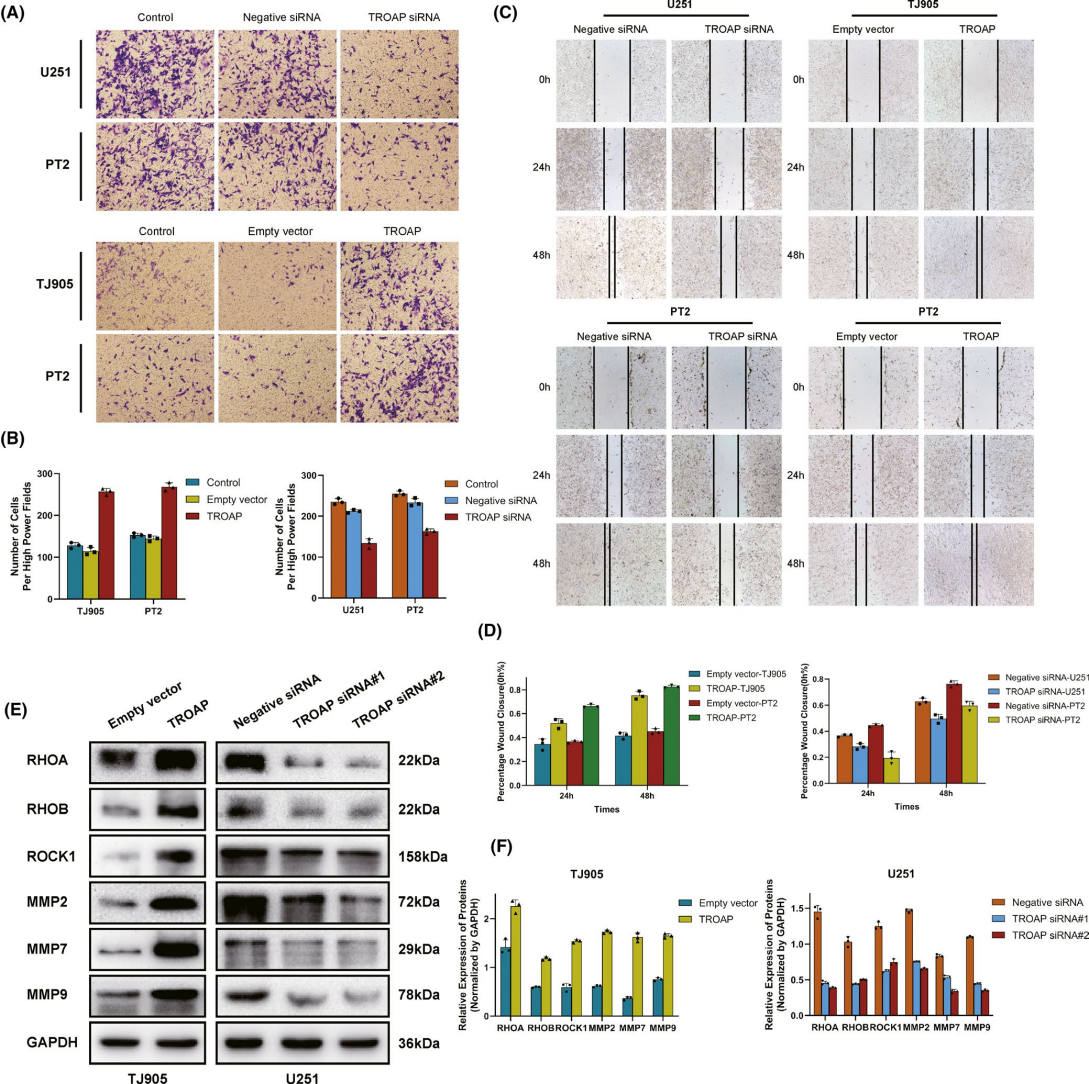
Upregulated TROAP promoted glioma cells invasion and migration in vitro. (A, B) Transwell assay to access the effects of TROAP overexpression or knockdown on cell migration and invasion in U251, TJ905 and PT2 cells. (C, D) Wound healing assay, images of the glioma cells after scratching 0h, 24h, 48h are shown in C and percentages of wound closure were shown in D. (E) Western blot assay to determine the expression changes of invasion markers in TROAP‐silencing/overexpressing TJ905 and U251 cells. Quantification graphs were shown in F (*p < 0.05, **p < 0.01, ***p < 0.001, respectively)

The role of TROAP in cell invasion and migration was explored by Transwell invasion and wound healing assays. Findings showed that U251 cells had attenuated invasion ability following transfection with TROAP‐siRNA, whereas the capability was increased after being transfected with TROAP overexpression vector in TJ905. Similarly, PT2 glioma cells also exhibited enhanced migration and invasion capability when treated with the TROAP overexpression plasmid compared to the empty vector, while attenuated migration was observed following transfection with TROAP siRNA (*p *< 0.05 Figure 4A–D). Then, the effect of TROAP expression on migration‐associated genes was examined by western blot analysis. The results revealed that knockdown of TROAP drastically downregulated the level of MMP2, MMP7, MMP9, RHOA, RHOA, ROCK1 protein in U251 glioma cell lines compared to control group (*p *< 0.05). While TROAP overexpression markedly elevated the expression of aforementioned migration‐related genes in TJ905 cells compared to control (Figure 4E,F). These data above indicated that TROAP‐induced migration and invasion in glioma cells via regulating the expression of migration‐related proteins.

### 3.5 TROAP facilitated gliomagenesis via activating Wnt/β‐Catenin signaling pathway

**FIGURE 5:**
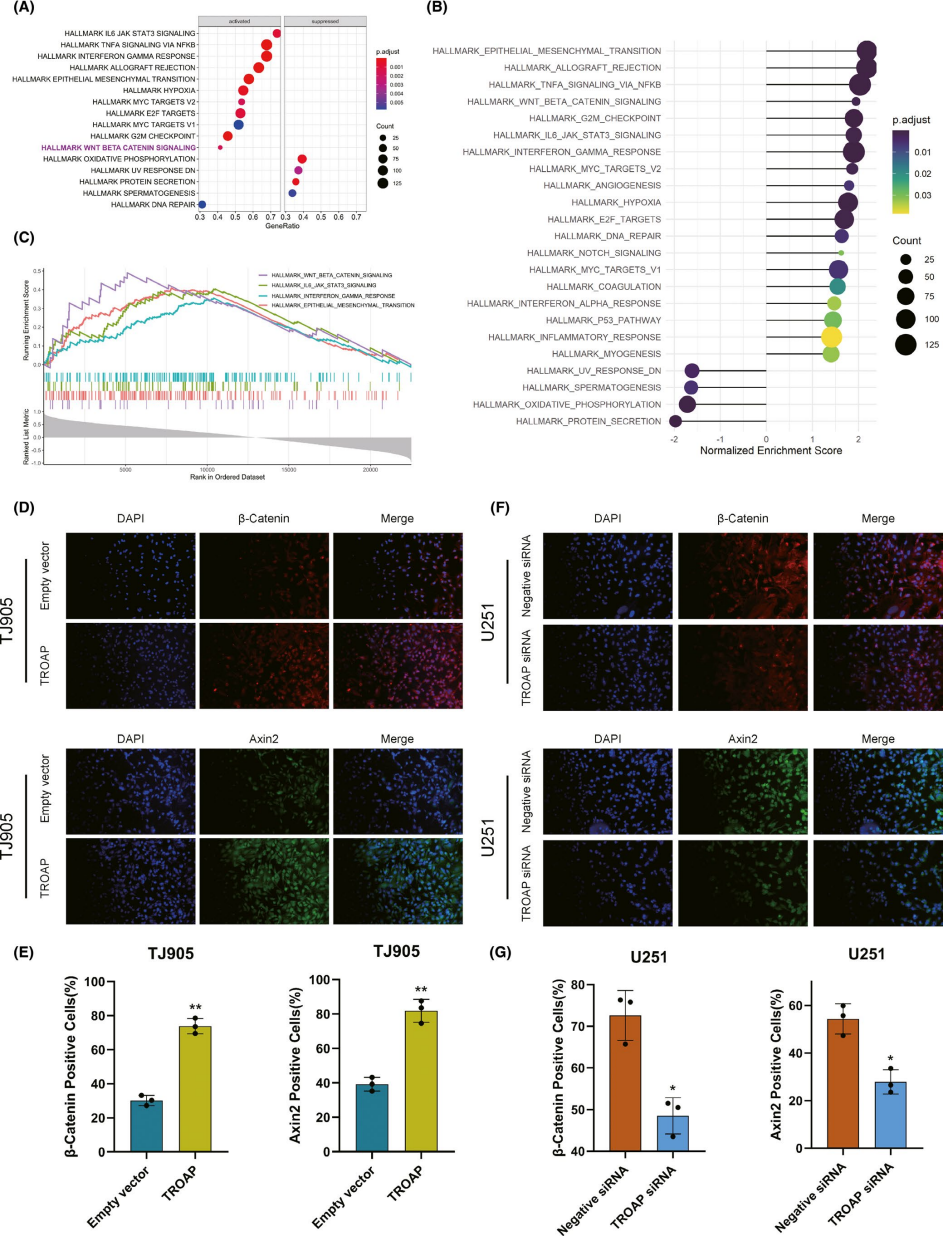
GSEA enrichment analysis of TROAP using the GEO database. (A, B) KEGG pathways were screened with the Fold change value greater than 0 and *p *< 0.05 as screening conditions. The dotplot showed the 19 upregulated (NES > 0) and 4 downregulated (NES < 0) pathways. (C) GSEA enrichment plots represented that enrichment of Wnt/β‐Catenin pathway. (D, E) Immunofluorescence showing overexpression of TROAP increased level of Axin2 protein in the cytoplasm of TJ905 cells compared to control, meanwhile, TROAP overexpression also upregulated the accumulation of β‐Catenin in the cytoplasm and nucleus of TJ905 cells compared to control. Immunofluorescence quantification analysis was shown in E. (F, G) Immunofluorescence assay on U251 cells transfected with TROAP siRNA (*p < 0.05, **p < 0.01, ***p < 0.001, respectively)

**FIGURE 6:**
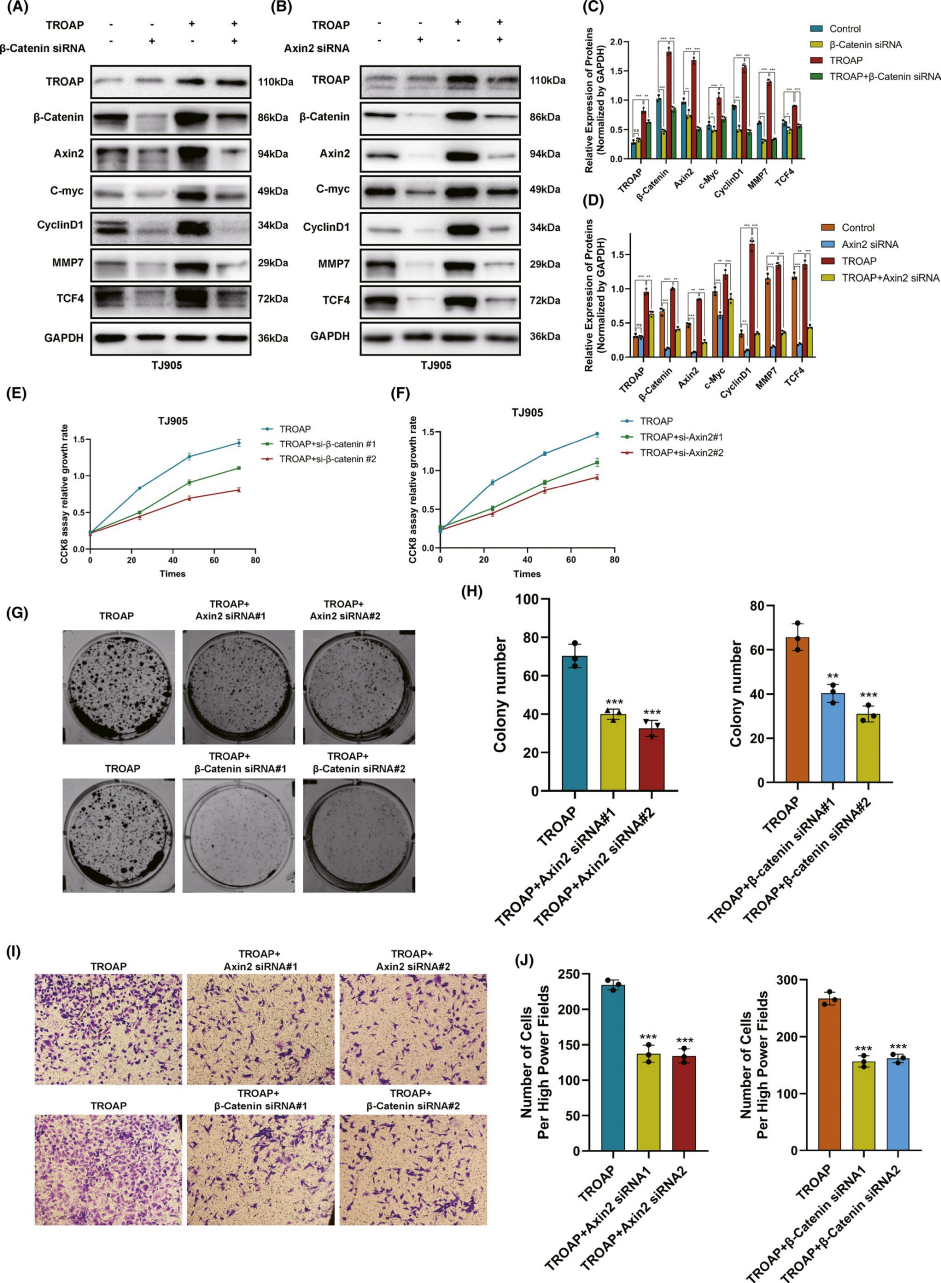
TROAP‐induced malignant phenotype via activating the Wnt/β‐Catenin signaling in glioma cells. (A, B) Western blot analysis showing the expression of Wnt/β‐Catenin signaling‐related proteins after silencing Axin2 or β‐Catenin in TJ905 cells ectopically overexpressing TROAP. The statistics were shown in the histogram C and D. The effect of TROAP overexpression on cell proliferation(E‐H), migration and invasion (I, J) was partly eliminated by silencing Axin2 or β‐Catenin (*p < 0.05, **p < 0.01, ***p < 0.001, respectively)

The molecular mechanisms in the process of TROAP‐induced oncogenic phenotype were evaluated. Firstly, the results of GSEA enrichment analysis identified 23 TROAP‐associated significantly enriched pathways (adj.*p*.value < 0.05, Figure 5A–B), including 19 activated pathways (normalized enrichment score, NES > 0) and 4 inactivated pathways (NES < 0). As shown in Figure 5C, the Wnt/β‐Catenin belonged to the activated signaling pathway. To our knowledge, Axin2 and β‐Catenin were two crucial regulatory molecular involved in Wnt/β‐Catenin pathway. Immunofluorescence (IF) assay was used to detect the levels of β‐Catenin and Axin2 in TJ905 cells transfected with TROAP overexpression vector. Findings indicated that TROAP overexpression induced substantial accumulation of Axin2 in cytoplasm and β‐Catenin in nucleus and cytoplasm of TJ905 cells compared to control (*p *< 0.05, Figure 5D–E). The results of IF in U251 cells transfected with TROAP siRNA showed that the intracellular fluorescence intensity of Axin2 and β‐Catenin was attenuated (*p *< 0.05, Figure 5F–G). The results of western blot analysis illustrated that TROAP overexpression could significantly upregulate the level of downstream target genes of Wnt/β‐Catenin signaling, such as Axin2, β‐Catenin, MMP7, C‐myc, CyclinD1 and TCF4 in TJ905 cells compared to control (*p *< 0.05, Figure 6A–D). While the expression of these proteins and TROAP‐induced tumor‐promoting effects (proliferation, migration and invasion) would be significantly reversed by silencing Axin2 or β‐Catenin (Figure 6E–J). Taken together, our findings indicated that TROAP‐induced malignant phenotype and tumorigenesis, at least in part, via activating Wnt/β‐Catenin signaling pathway.

## 4 DISCUSSION

TROAP, a soluble cytoplasmic protein, was found to display highly cooperative binding to trophinin and bystin at the interface of endometrium and trophoblasts by Fukuda.^9^ The associated adhesion binding complex functions in early embryo implantation were implicated by rapid cellular proliferation and invasion. In previous study, TROAP’s functions were only implicated in regulation of physiological process (embryo implantation, mitosis), until recent several studies pointed it out that TROAP was aberrantly upregulated in many types of clinically aggressive tumors. TROAP was reportedly increased in hepatocellular carcinoma (HCC) and correlated with the degree of malignancies as well as poor prognosis.^16^ Furthermore, TROAP was known to be highly expressed in gallbladder cancer tissue and promoted tumor invasion and metastasis.^21^ Concordant with these evidence, silencing TROAP could induce significantly attenuated proliferation of Hela cell lines.^11^ While, studies on its potential functional mechanism in glioma were elusive. In the present study, mRNA and protein expression of TROAP was significantly increased in glioma tissues and cell lines, TROAP overexpression was correlated with lower survival rates, which also proved the results of bioinformatic analysis. Therefore, we speculated that the downregulation of TROAP might be a potential strategy for treating glioma. Reorganization of cytoskeleton in the essential mechanism underlied the cell motility. TROAP has been validated as a MT‐associated protein that maintained the dynamic feature of centrosome, contributing to normal structural function.^22^, ^23^ Crosstalk between microtubules and cytoskeleton could result in cellular invasion and migration, a process crucial for tumorigenesis and malignant phenotype.^24^ In our study, the effect of TROAP on biological behavior of glioma cells was investigated. As indicated by western blot, TROAP downregulation using siRNA transfection could result in markedly attenuated cell proliferation, migration and invasion, suggesting that TROAP expression was correlated with the malignant potential of glioma. Deregulated growth was an essential requirement for the tumorigenesis and was closely related with dysregulation of cell cycle.^25^ TROAP severed as a cycling protein essential for mitosis and its endogenous expression was tightly controlled during cell cycle progression. Besides, TROAP contained several sites for serine/threonine phosphorylation of multiple protein kinases, such as mitogen‐activated protein kinases and cyclin‐dependent kinase.^26^ In line with the notions above, our study found that lower level of TROAP used siRNA transfection significantly inhibited cell viability of U251 cell lines by regulating G1/S cell cycle transition, suggesting that TROAP overexpression might induce genomic imbalances and contribute to gliomagenesis.

Studies showed that Wnt/β‐Catenin signaling pathways played a crucial role in the physiological behavior, disease and tumorigenesis, importantly, the abnormal activation and mutation of Wnt/β‐Catenin pathway were closely related to the gliomagenesis.^27^, ^28^, ^29^ Canonical Wnt signaling pathway was controlled by β‐Catenin, whose degradation was mediated by Axin2.^30^ Hence, our immunofluorescence assay showed that TROAP overexpression resulted in the elevated Axin2 and β‐Catenin level, while these genes were downregulated once TROAP silenced, suggesting that TROAP might be an upstream positive regulator in Wnt/β‐Catenin signaling pathway. We hypothesized that aberrantly upregulated TROAP might prevent β‐Catenin from being degraded by Axin2, leading to the substantial accumulation of β‐Catenin in cytoplasm and then transportation into nucleus, where it activated the downstream target genes to activate Wnt/β‐Catenin signaling, inducing tumorigenesis.^31^, ^32^, ^33^ Furthermore, gene set enrichment analysis (GSEA) analysis uncovered similar results. To get more accurate molecular mechanism, accordingly, our study reported that TROAP overexpression increased the level of two key pathway regulatory factors, including β‐Catenin and Axin2, and a series of downstream targets proteins (C‐myc, CyclinD1, MMP7 and TCF4). Conversely, the phenomenon would be partly reversed by silencing Axin2 and β‐Catenin.

In conclusion, our study revealed that TROAP could promote malignant phenotype of glioma cells in vitro, at least in part, activating Wnt/β‐Catenin signaling pathway, thus proving the role of TROAP and underlying molecular mechanism of TROAP in gliomagenesis for the first time. We hope our findings on TROAP‐induced glioma progress will provide new clues for guiding therapeutic strategies in glioma.

## CONFLICT OF INTEREST

No potential conflicts of interest were disclosed.

## Supporting information

Supplementary Material

## Data Availability

The original contribution presented in the study are included in the article/supplement material. Future inquiries can be directed to the corresponding authors.
