# Supplementary material for: TROAP regulates cell cycle and promotes tumor progression through Wnt/β‐Catenin signaling pathway in glioma cells
Source: CNS Neurosci Ther. 2021 Jun 2;27(9):1064–76. doi: 10.1111/cns.13688 (PMC8339535; doi:10.1111/cns.13688)
Supplement: Supplementary file 1 — Supplementary Material [file CNS-27-1064-s001.pdf]

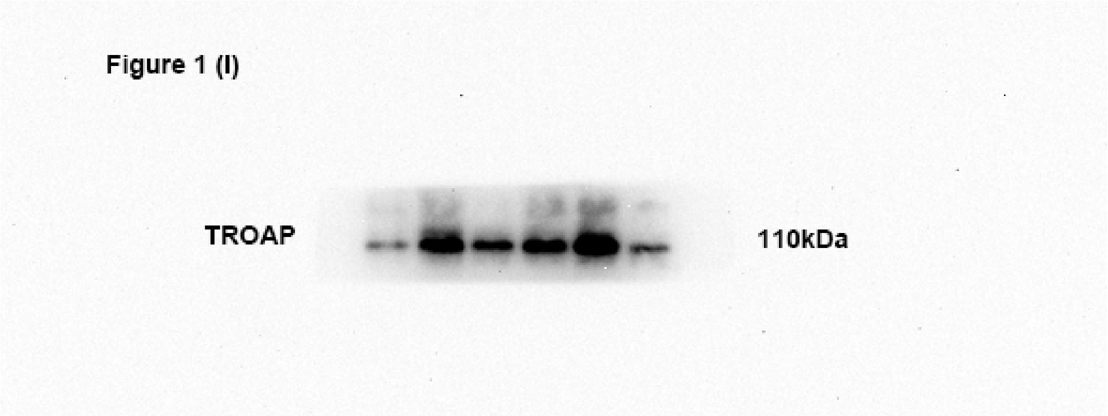

Figure1 (I)

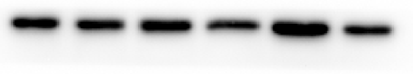

Figure1（G）

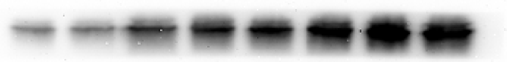

GAPDH 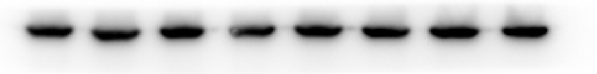 36kDa

Figure3(A)

TROAP 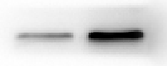 110kDa

Figure3A

GAPDH 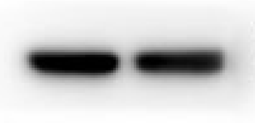 36kDa

Figure3B

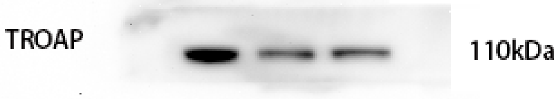

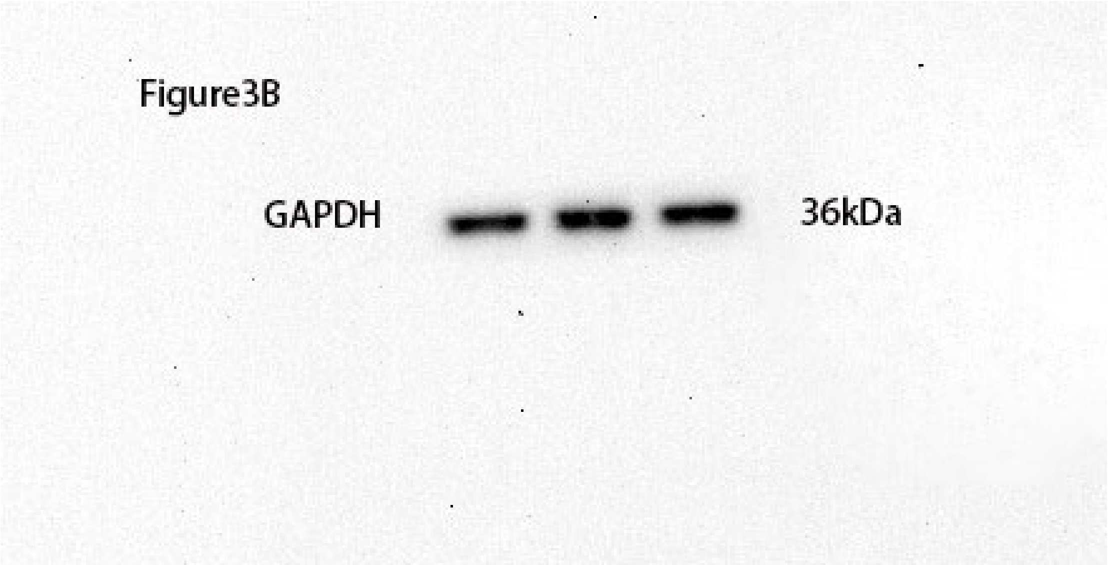

Figure3C

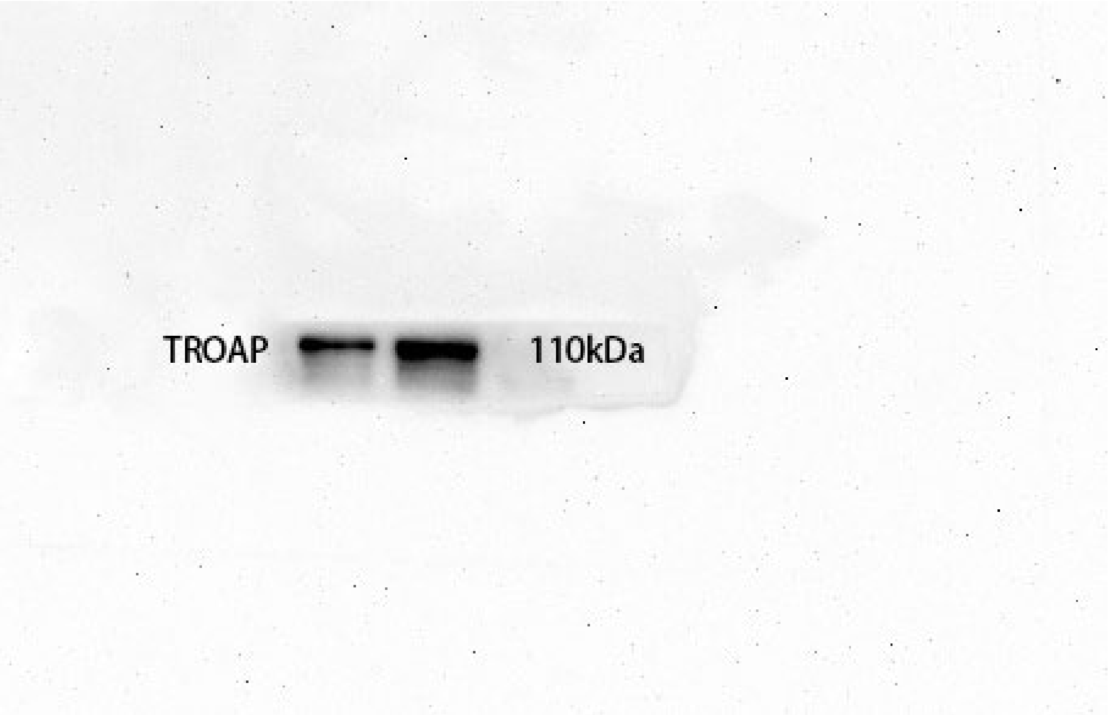
TROAP
110kDa

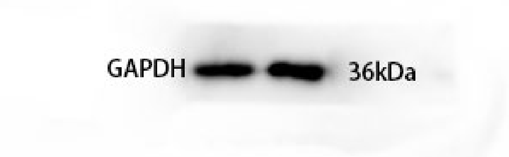
GAPDH
36kDa

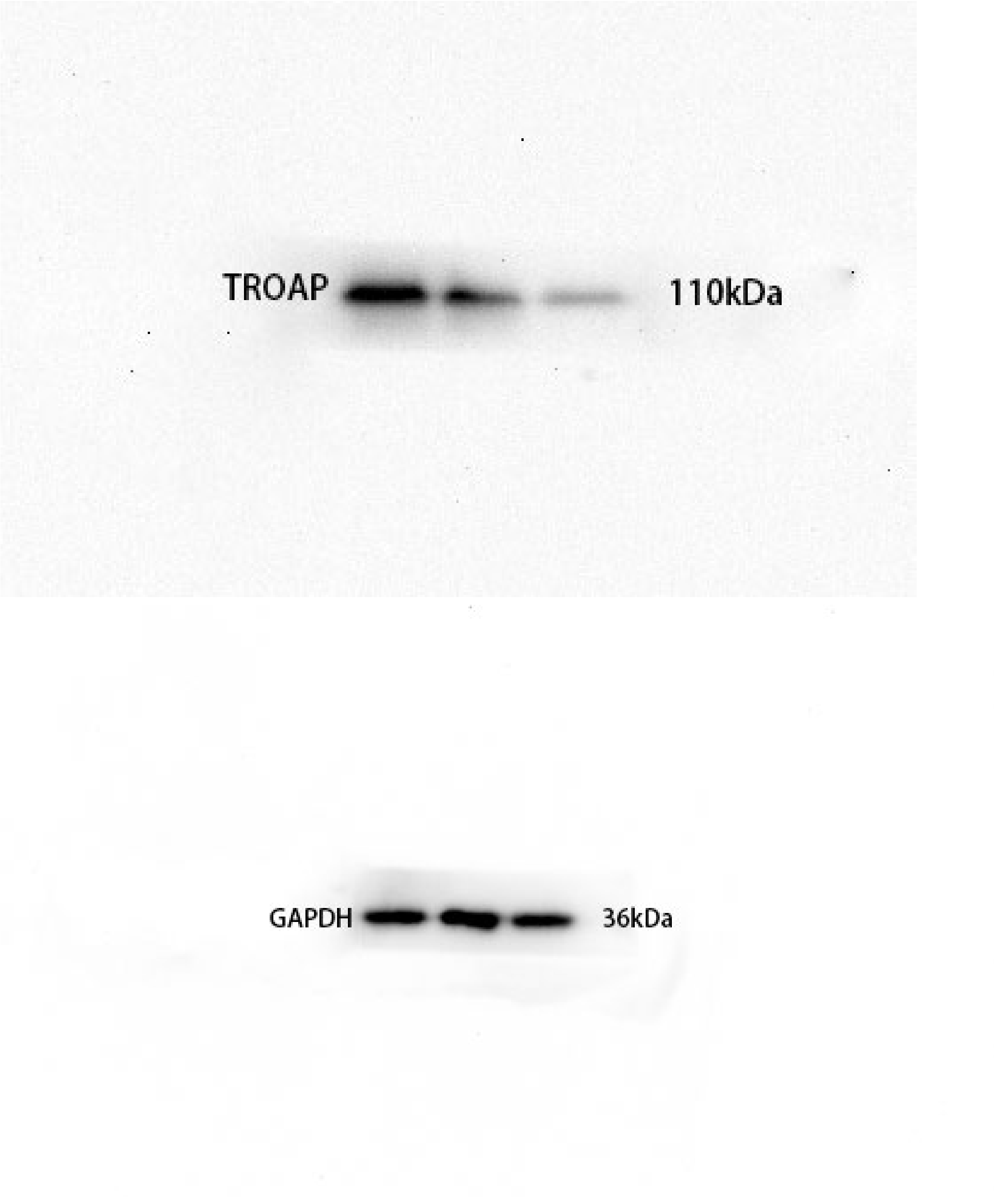

Figure4E

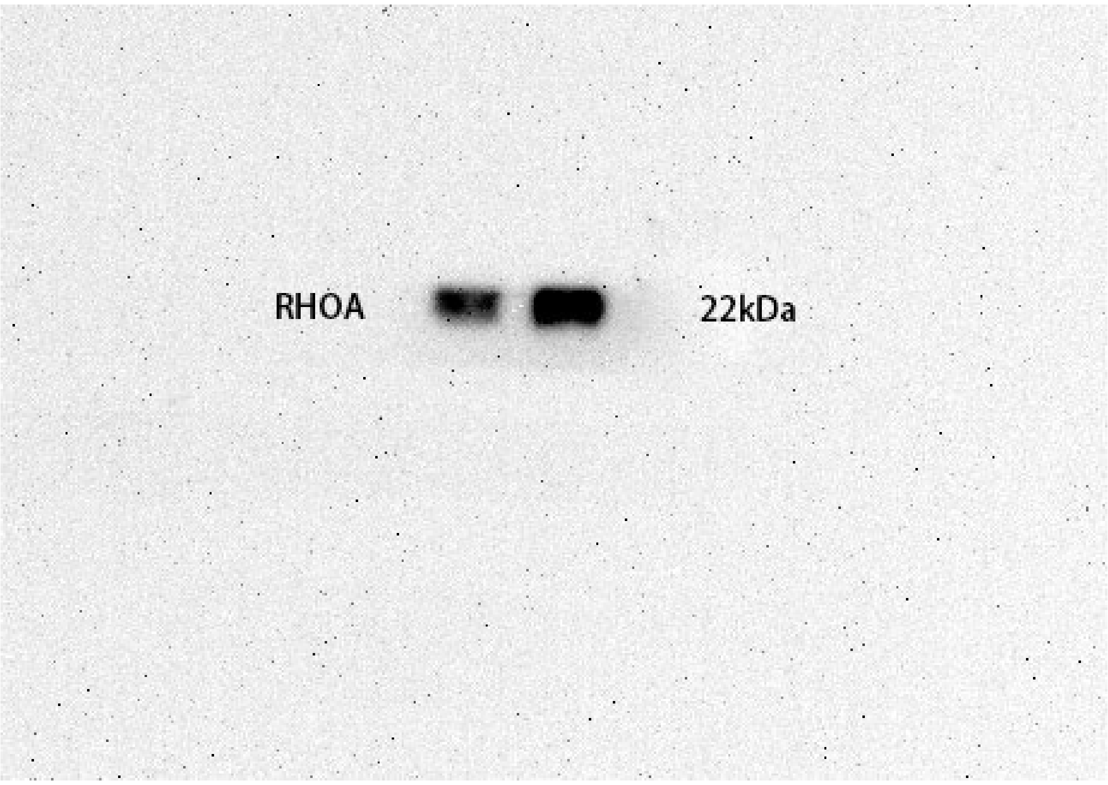
RHOA
22kDa

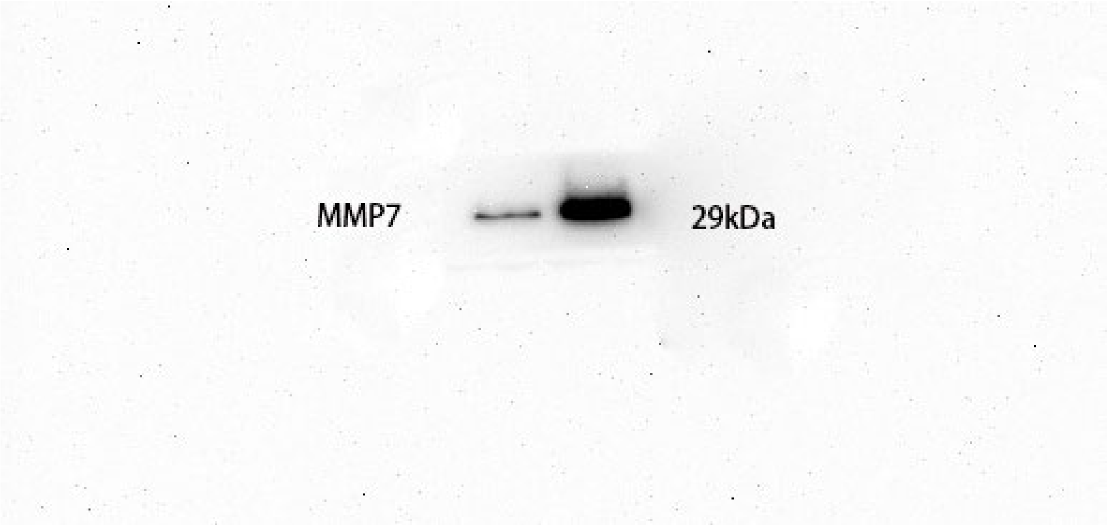
MMP7
29kDa

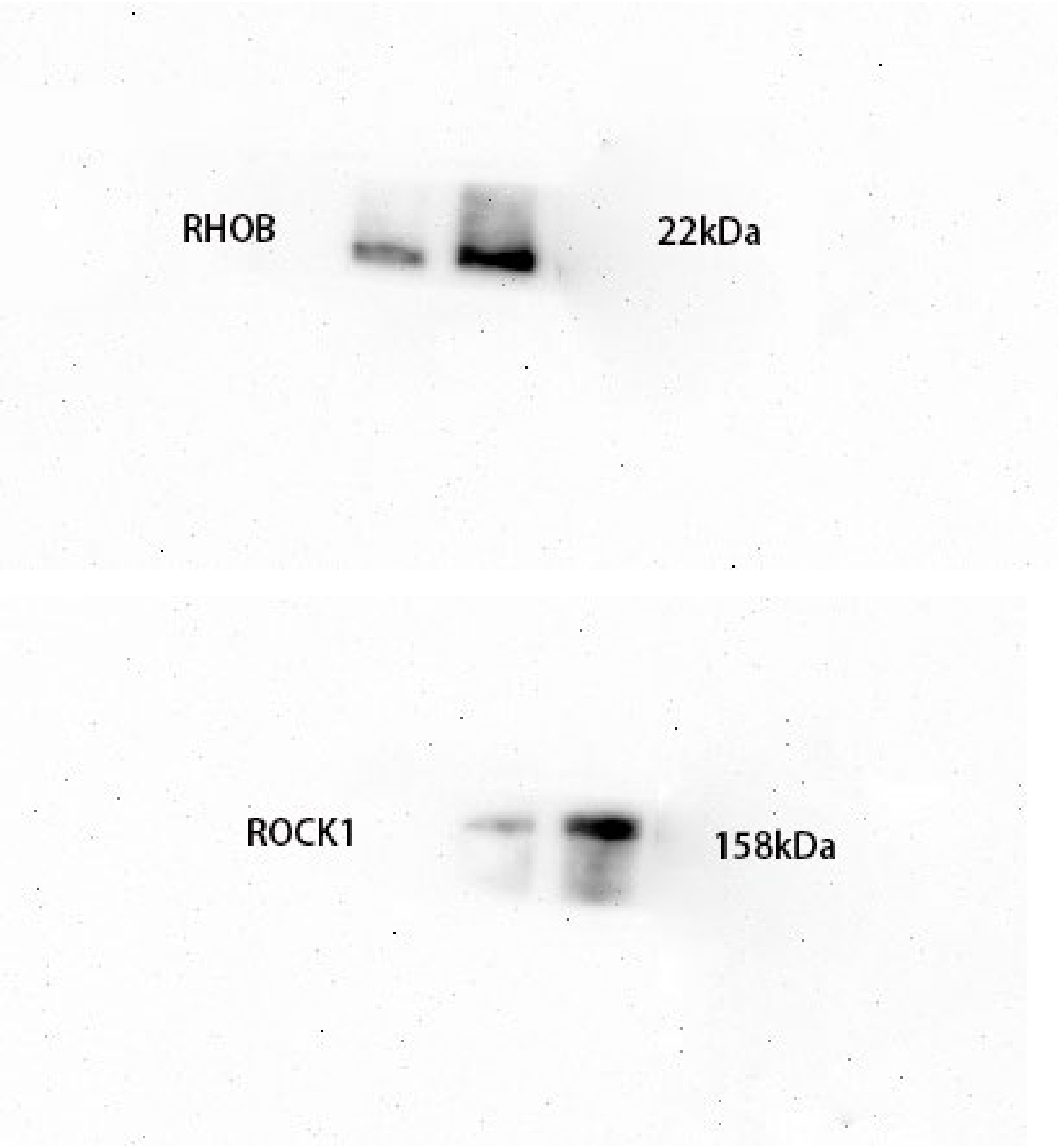
RHOB
22kDa
ROCK1
158kDa

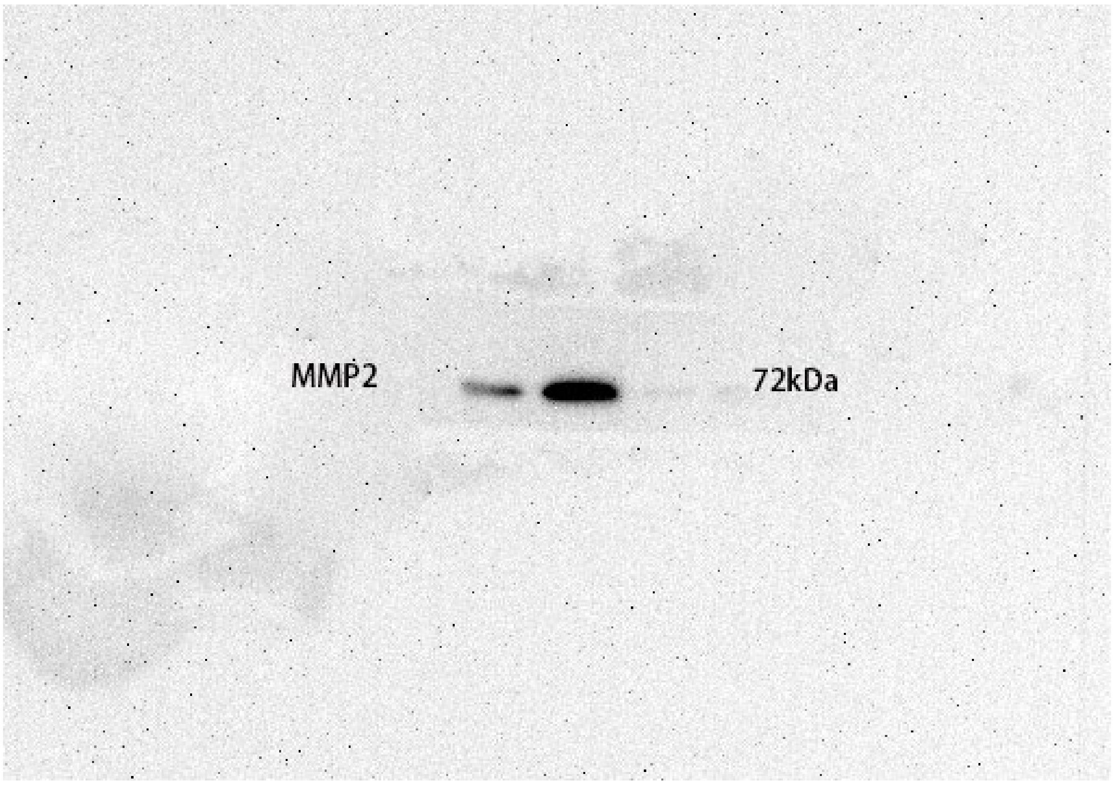
MMP2
72kDa

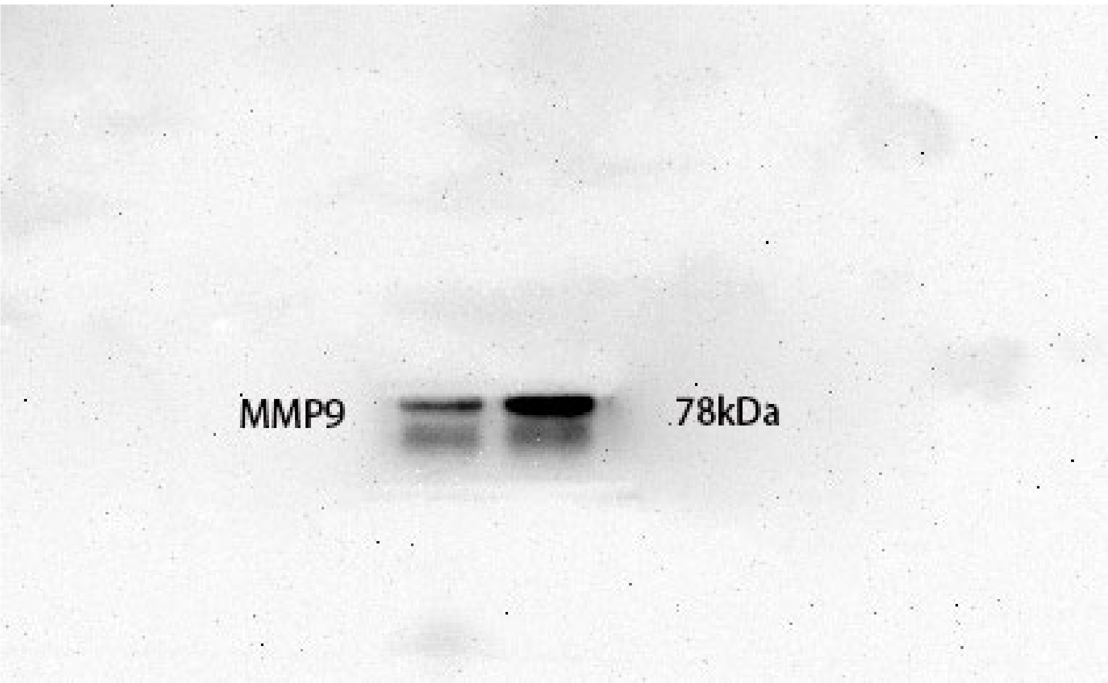
MMP9
78kDa

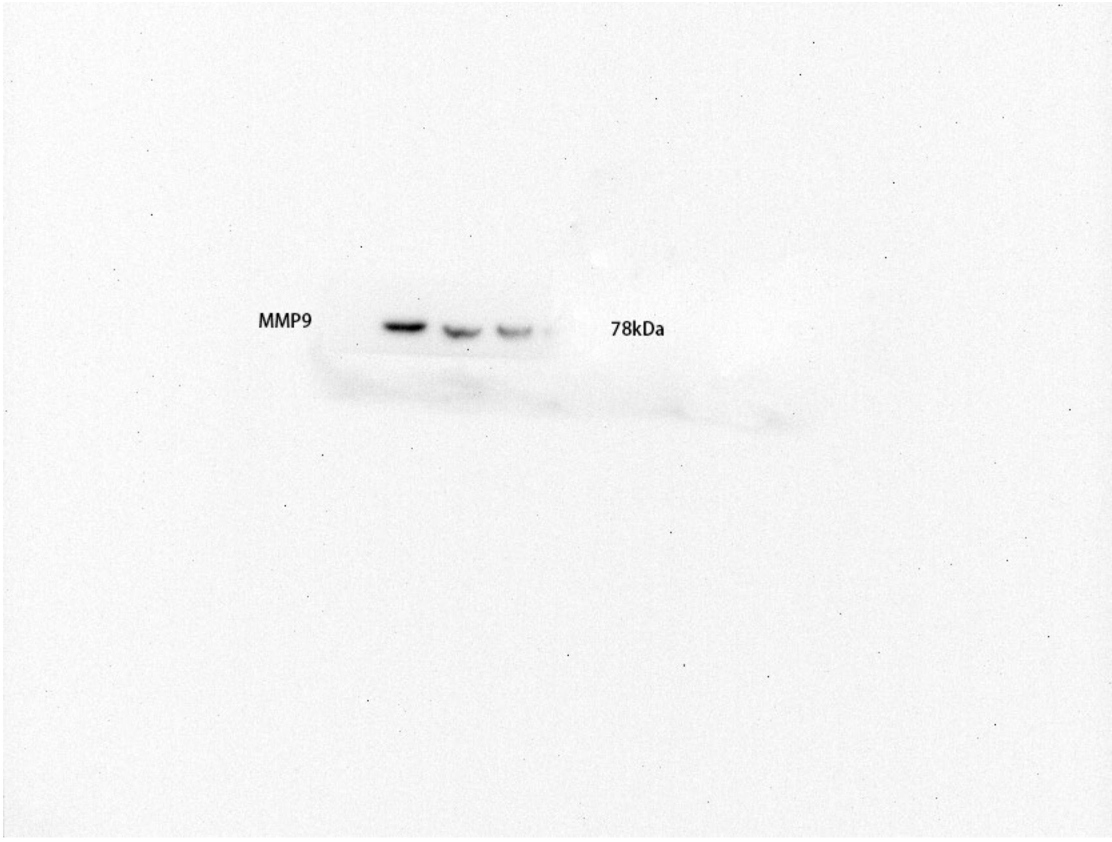
MMP9
78kDa

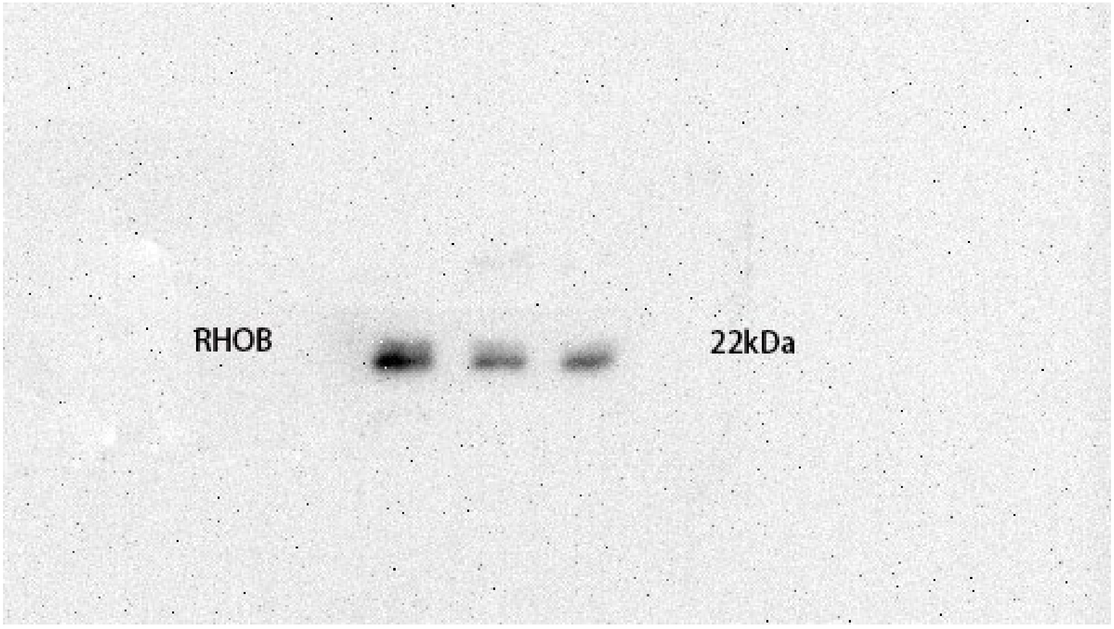
RHOB
22kDa

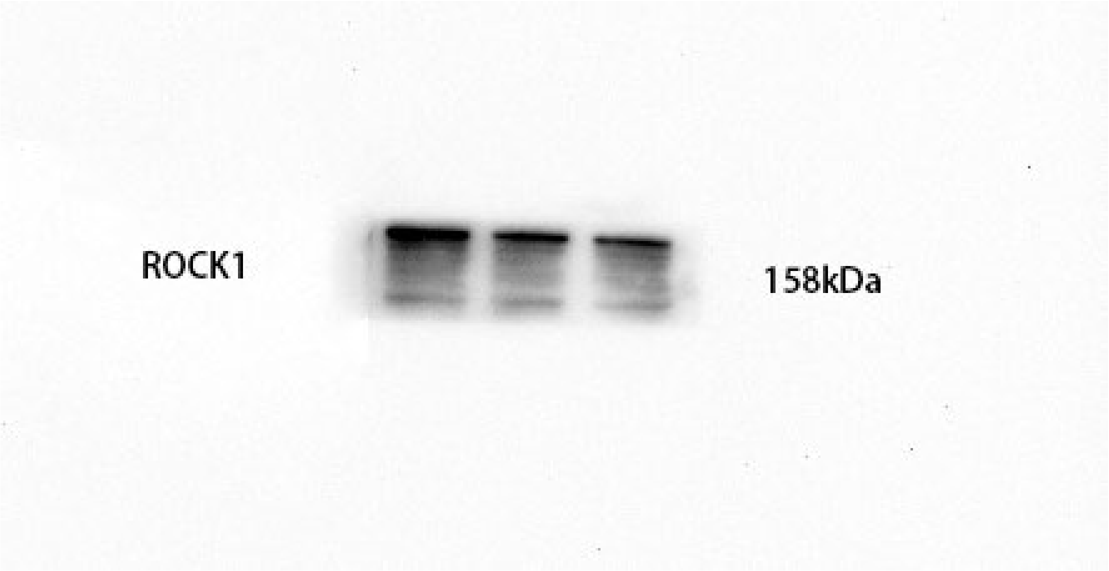
ROCK1
158kDa

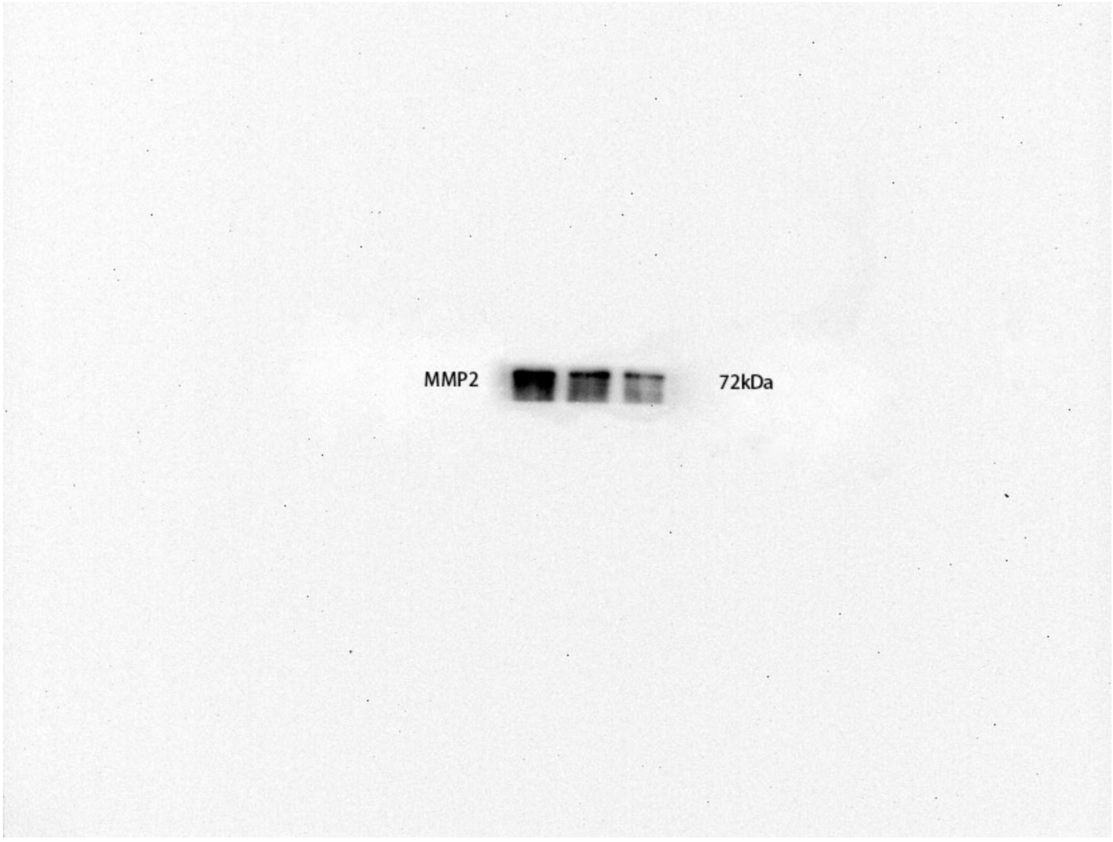
MMP2
72kDa

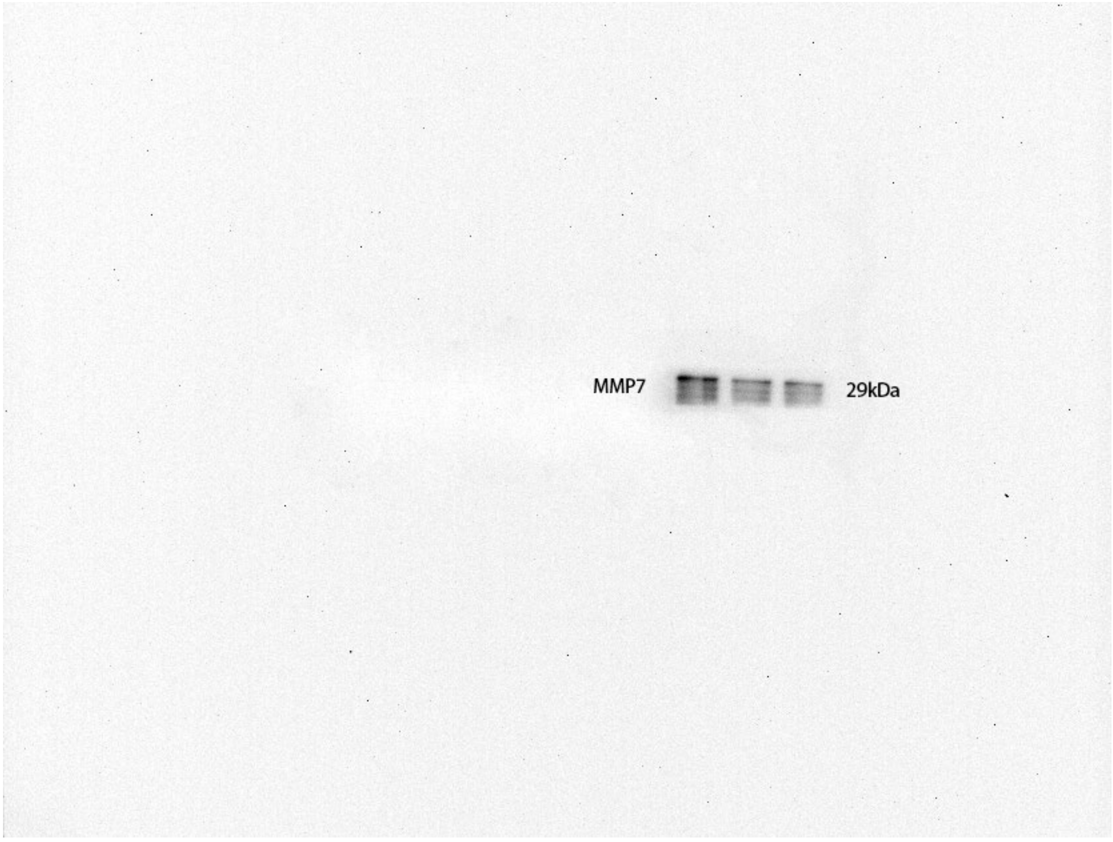
MMP7
29kDa

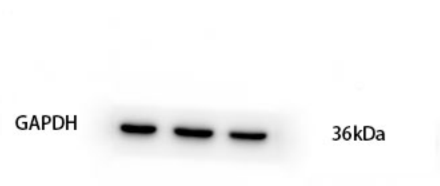
GAPDH
36kDa

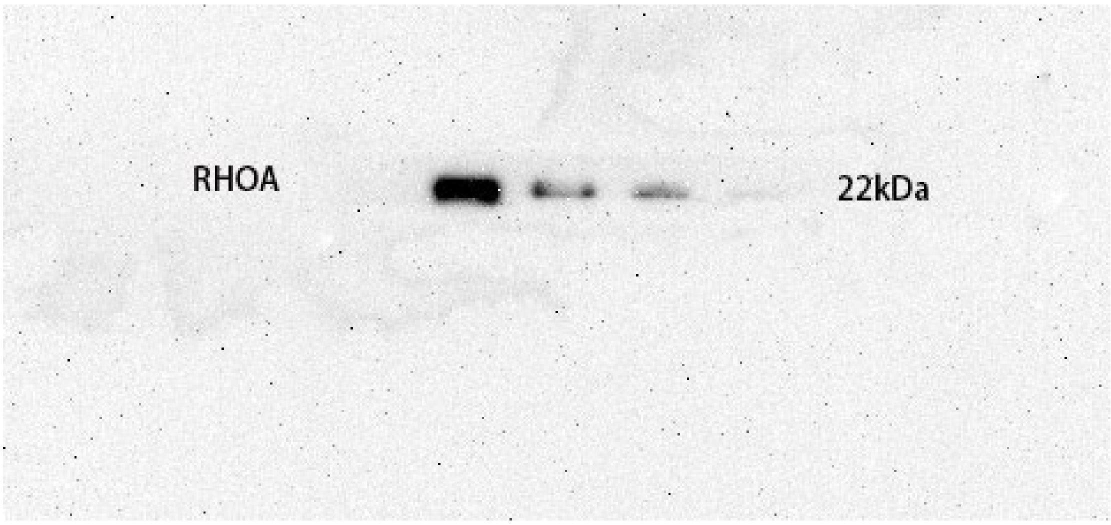

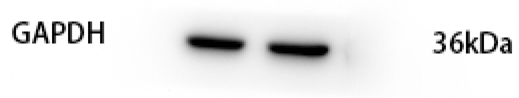

Figure6A

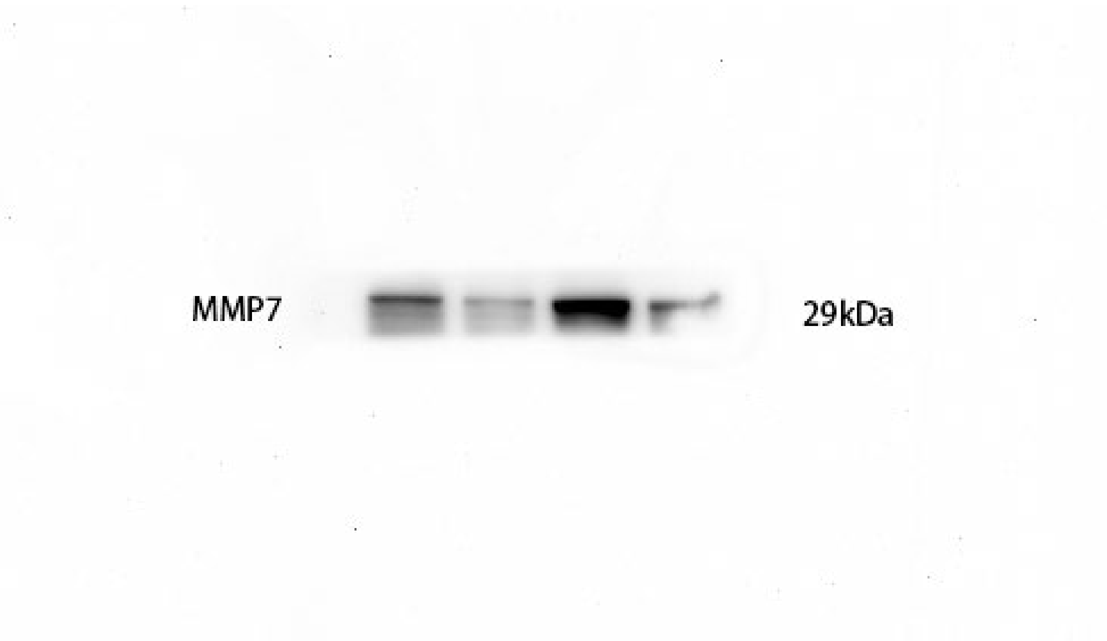

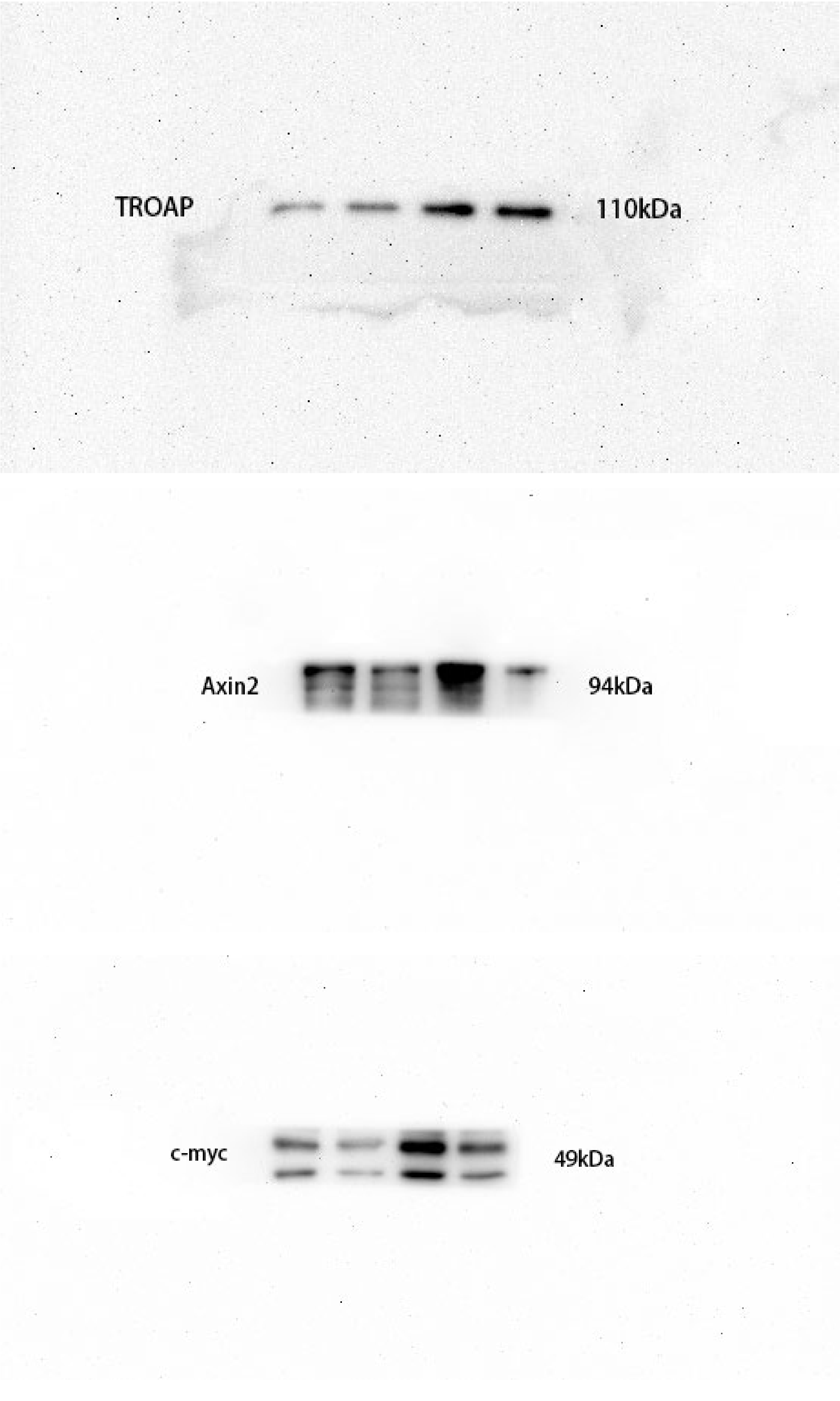
TROAP
110kDa
Axin2
94kDa
c-myc
49kDa

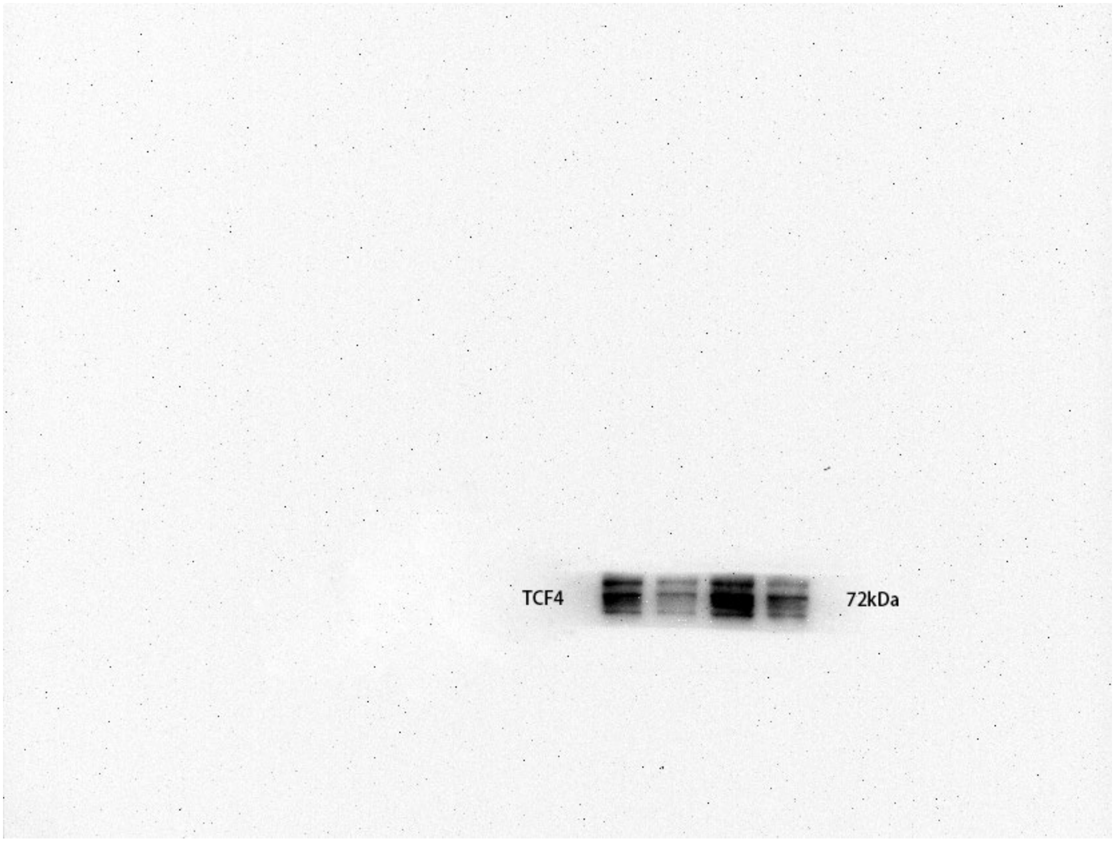

TCF4
72kDa

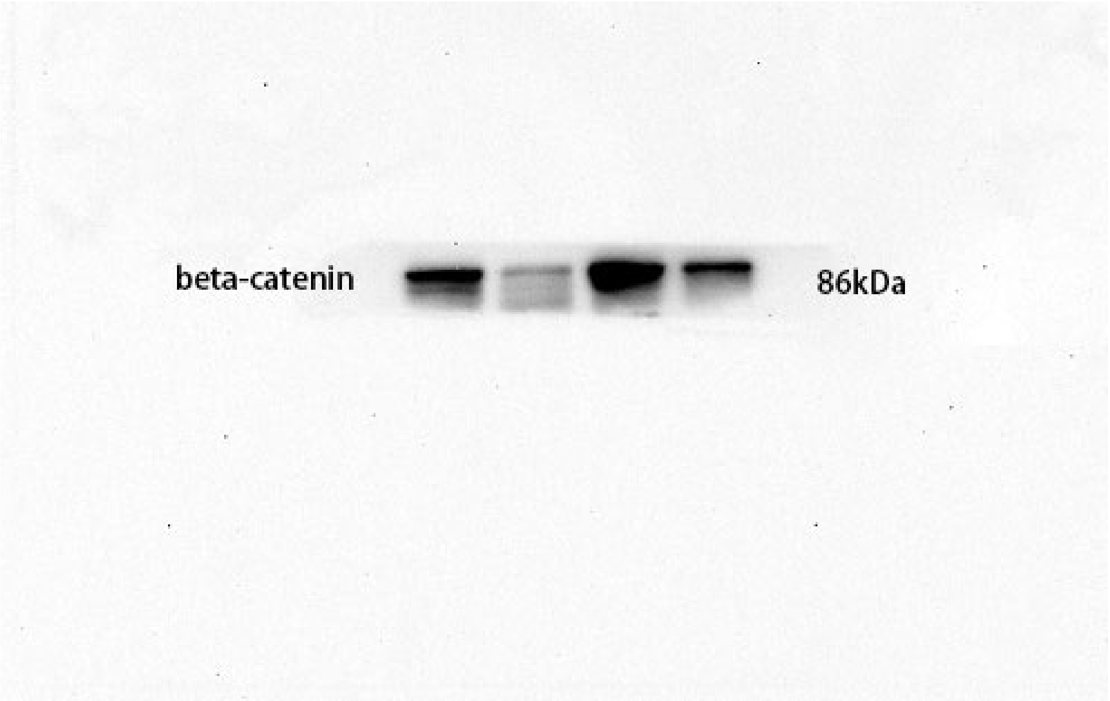

beta-catenin
86kDa

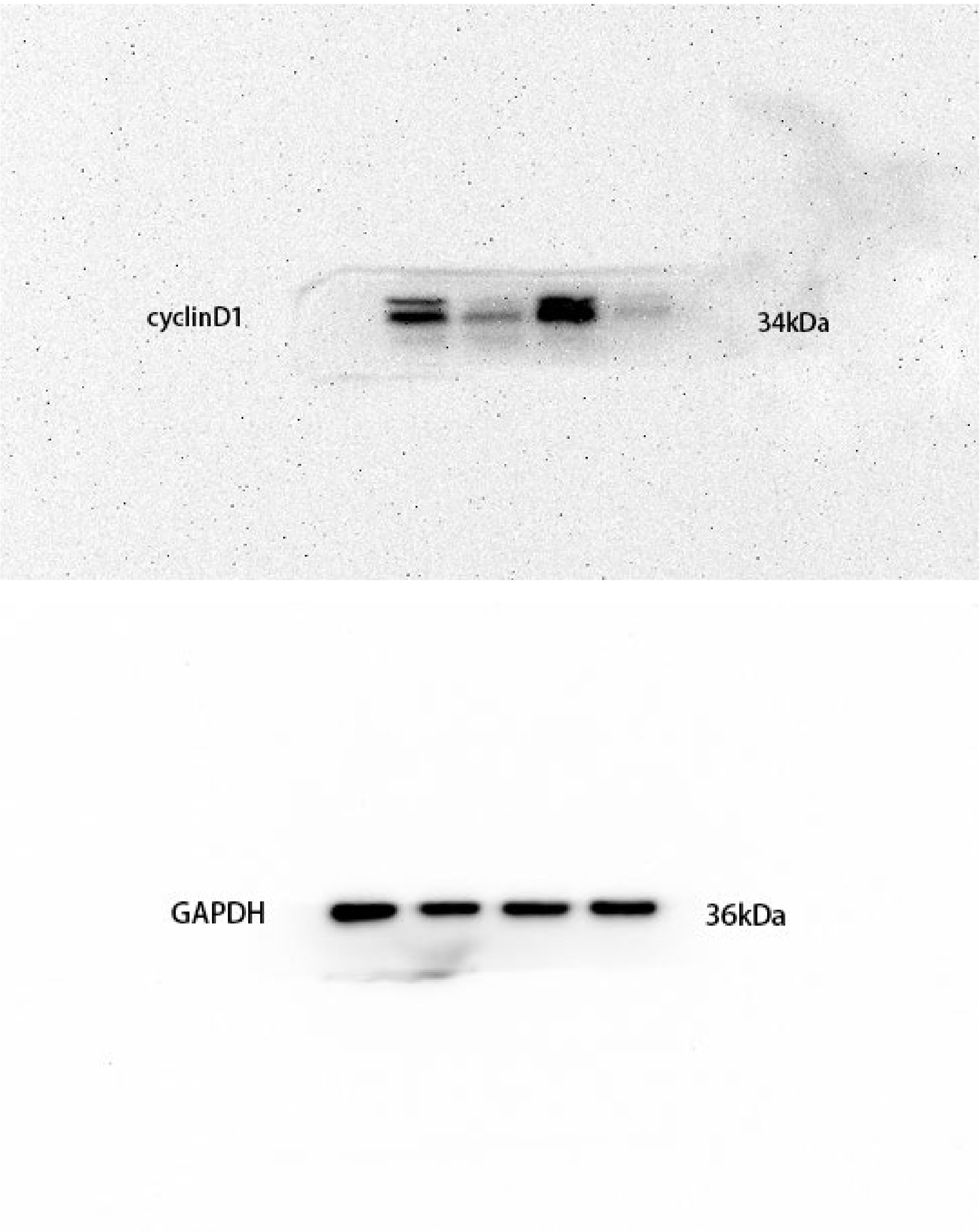
cyclinD1
34kDa
GAPDH
36kDa

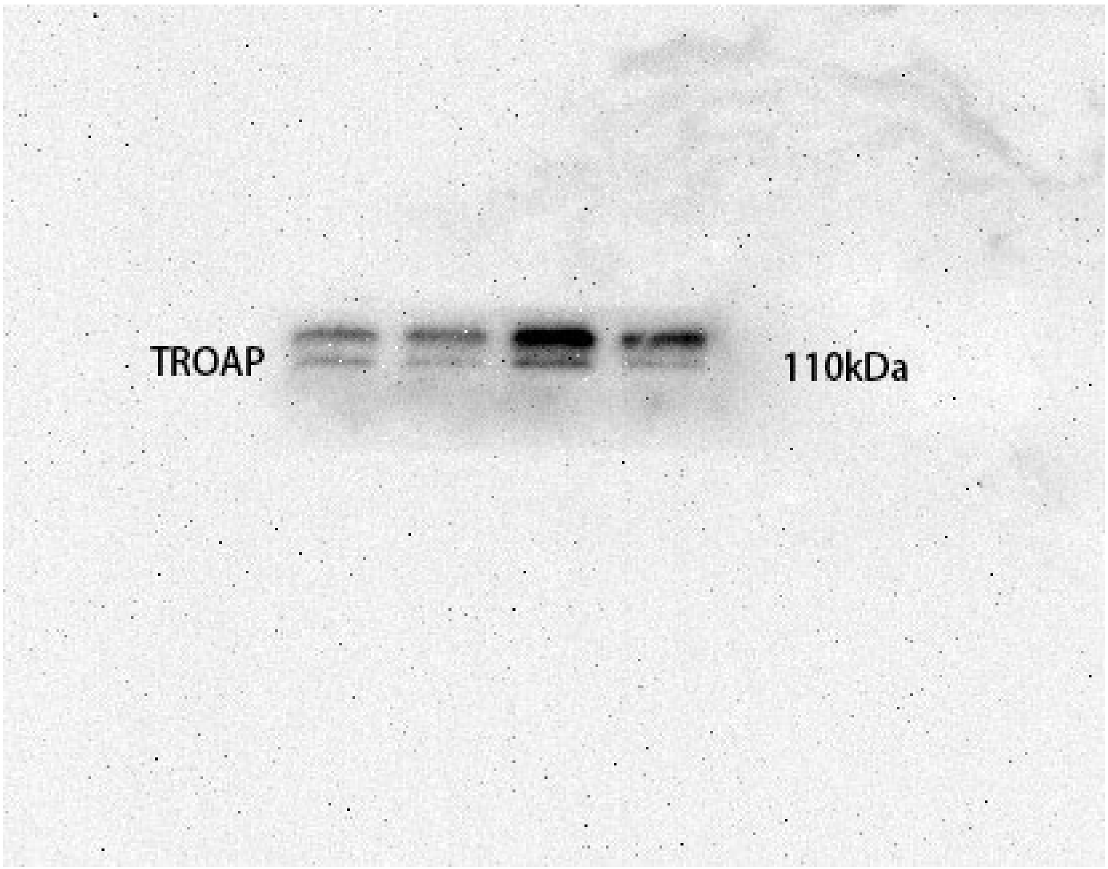
TROAP
110kDa

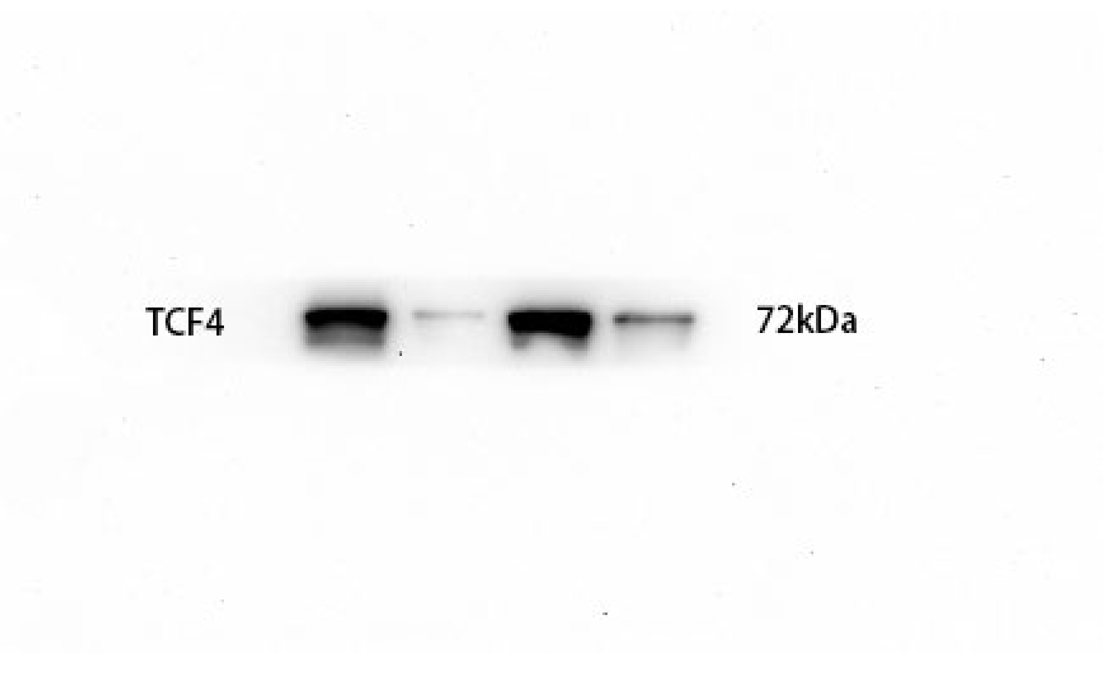
TCF4
72kDa

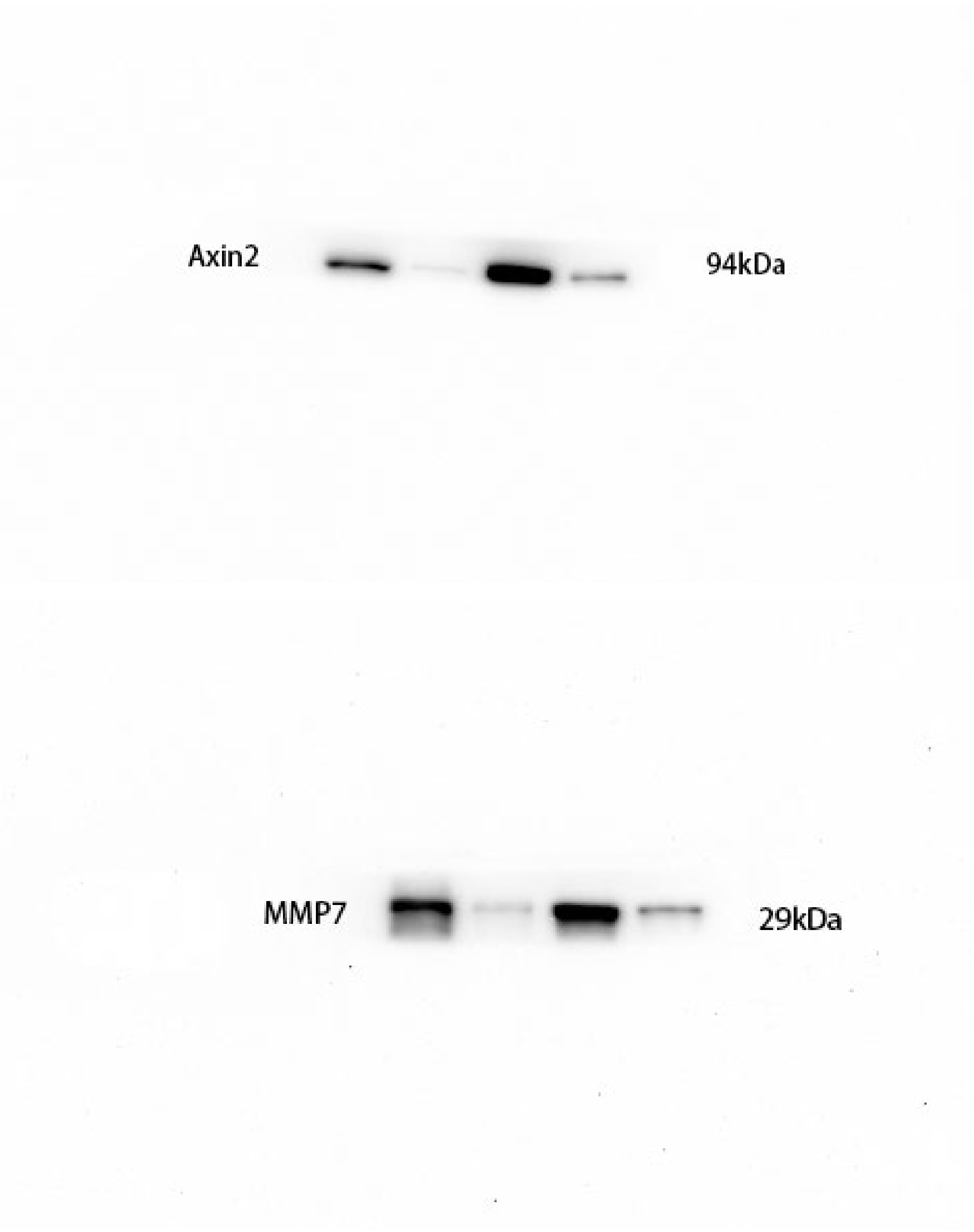
Axin2
94kDa
MMP7
29kDa

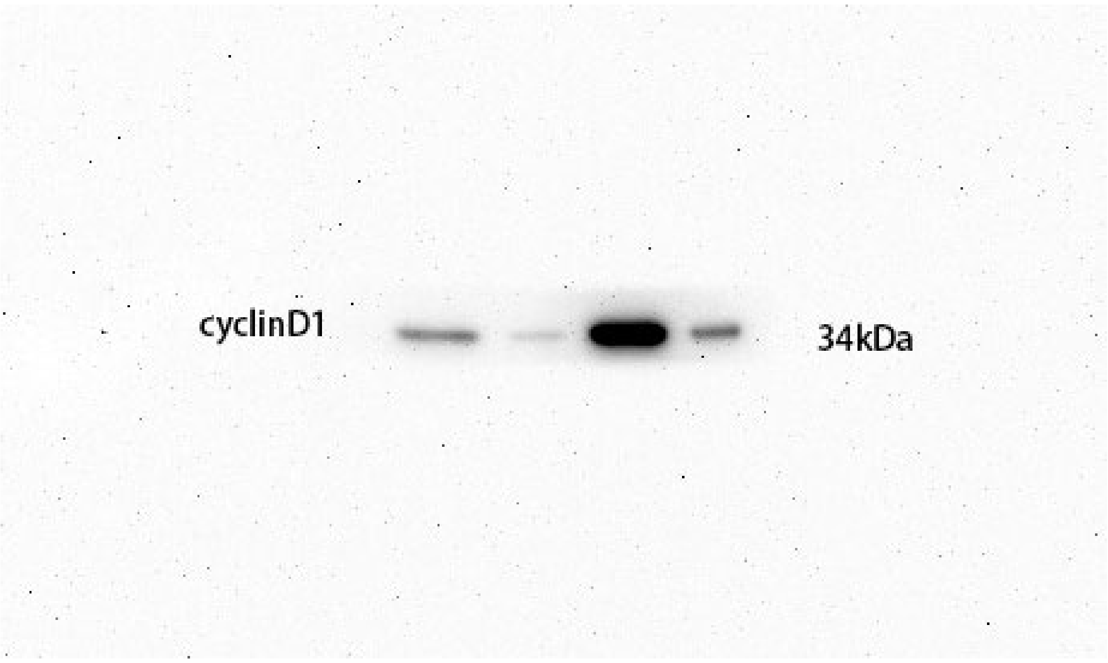
cyclinD1
34kDa

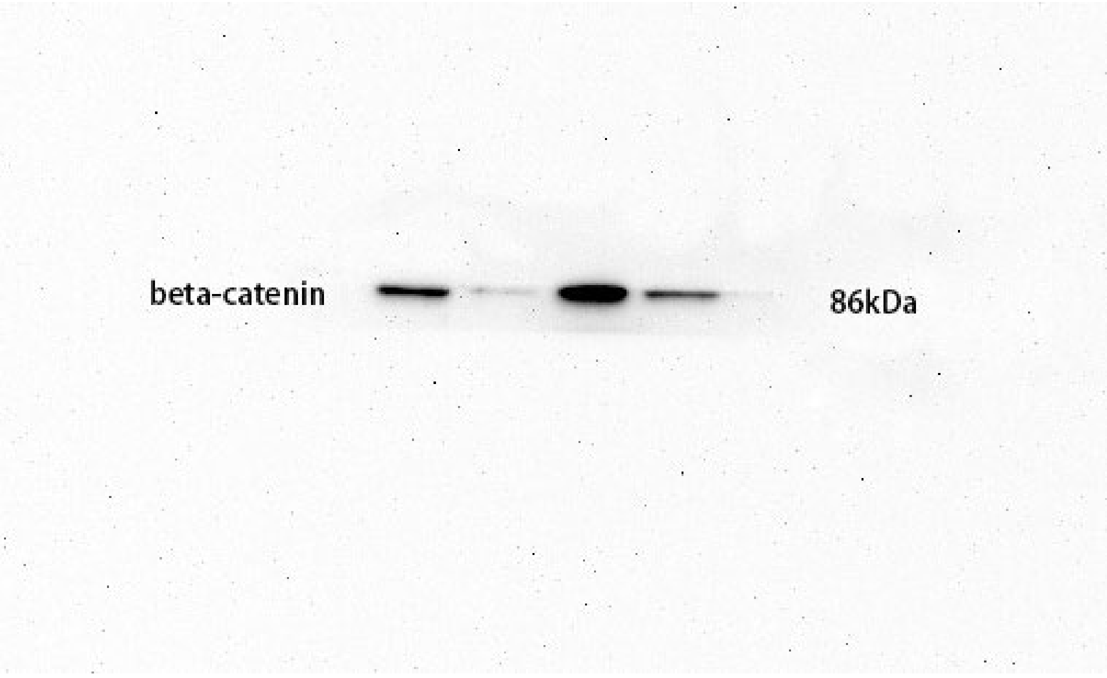
beta-catenin
86kDa

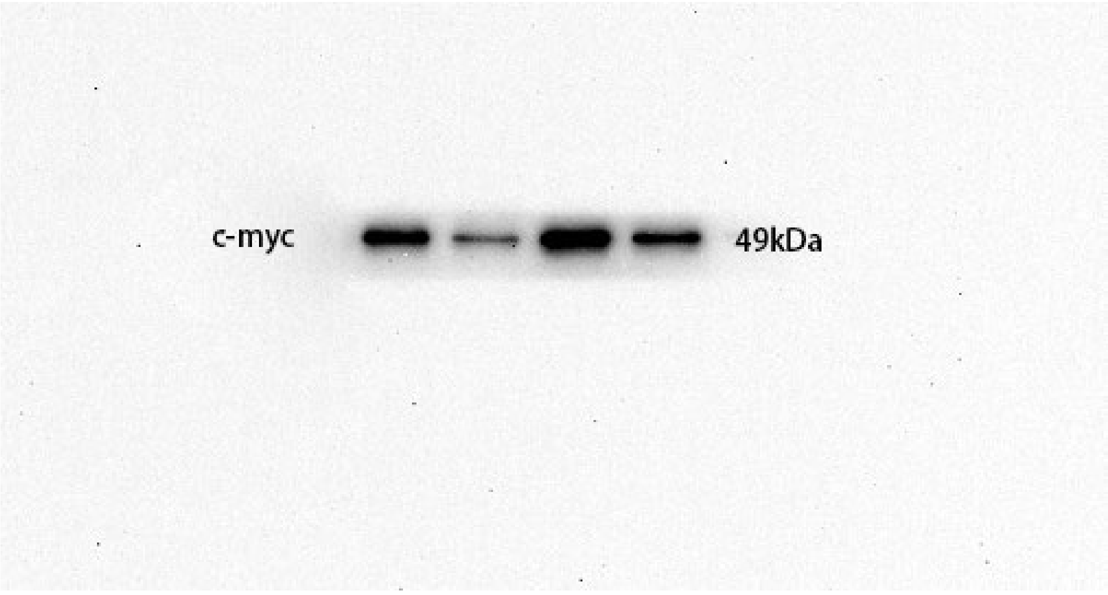
c-myc
49kDa

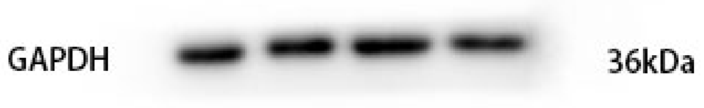
GAPDH
36kDa
